# Synthesis of compounds related to the anti-migraine drug eletriptan hydrobromide

**DOI:** 10.3762/bjoc.8.162

**Published:** 2012-08-30

**Authors:** Suri Babu Madasu, Nagaji Ambabhai Vekariya, M N V D Hari Kiran, Badarinadh Gupta, Aminul Islam, Paul S Douglas, Korupolu Raghu Babu

**Affiliations:** 1 Chemical Research and Development, Aurobindo Pharma Ltd., Survey No. 71 & 72, Indrakaran (V), Sangareddy (M), Medak Dist-502329, Andhra Pradesh, India; 2 Engineering Chemistry Department, AU College of Engineering, Andhra University, Visakhapatnam-530003, Andhra Pradesh, India

**Keywords:** characterization, control, eletriptan, origin, related substances

## Abstract

Eletriptan hydrobromide (**1**) is a selective serotonin (5-HT_1_) agonist, used for the acute treatment of the headache phase of migraine attacks. During the manufacture of eletriptan hydrobromide the formation of various impurities were observed and identified by LC–MS. To control the formation of these impurities during the preparation of active pharmaceutical ingredients, the structure of the impurities must be known. Major impurities of the eletriptan hydrobromide synthesis were prepared and characterized by using various spectroscopic techniques, i.e., mass spectroscopy, FTIR , ^1^H NMR, ^13^C NMR/DEPT, and further confirmed by co-injection in HPLC. The present study will be of great help in the synthesis of highly pure eletriptan hydrobromide related compounds.

## Introduction

**Figure 1:**
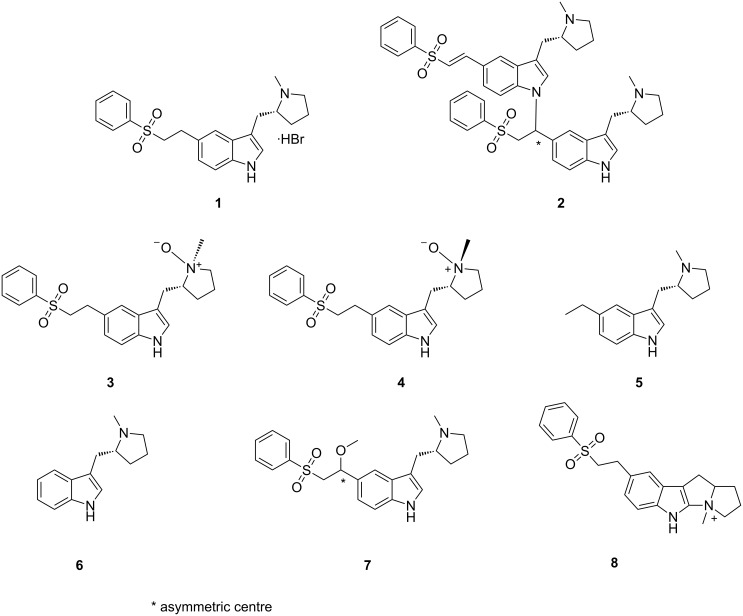
Related compounds of eletriptan hydrobromide.

Eletriptan hydrobromide was first disclosed in U.S. patent 5,545,644 (1996), assigned to Pfizer, New York, claiming the product “eletriptan” and its pharmaceutically acceptable salts thereof. An optimization of the eletriptan hydrobromide synthesis to prepare chemically pure eletriptan hydrobromide was reported in the literature [1–2]. However, a detailed study on the profile of the impurities present and their synthesis has not yet been cited anywhere, except for in the case of some metabolites [3]. Eletriptan hydrobromide (**1**, Figure 1) is a second-generation drug serotonin (5-HT_1_) agonist [4–5] used in the management of sensations of tightness, pain, pressure and heaviness in the precordium, throat and jaws. Eletriptan is more lipophilic than other triptans and absorbed more quickly than sumatriptan in the intestinal absorption. Eletriptan is more effective than sumatriptan in reducing the blood vessels surrounding the brain, which cause the swelling that is associated with the headache pain of a migraine attack, by blocking the release of substances from the nerve endings that causes more pain.

**Scheme 1:**
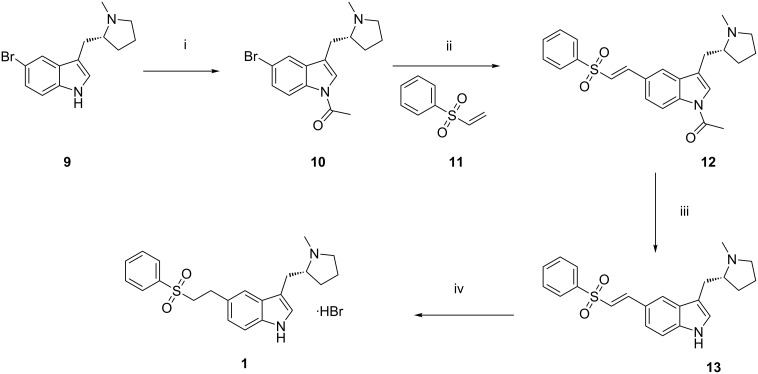
Synthetic route of eletriptan hydrobromide. Reagents and conditions: (i) Acetic anhydride, TEA, DMF, 90–100 °C; (ii) palladium acetate, tri-(*o*-tolyl)phosphine, TEA, DMF, 90–100 °C; (iii) methanol, K_2_CO_3_, acetonitrile, H_2_O, 5–10 °C; (iv) palladium on carbon, acetone, H_2_O, aqueous hydrobromic acid, IPA, 25–30 °C.

Eletriptan is metabolized by the enzyme CYP3A4 and designated chemically as (*R*)-5-[2(phenylsulfonyl)ethyl]-3-(*N*-methylpyrrolidin-2-ylmethyl)-1*H*-indole. Various procedures for the synthesis of eletriptan hydrobromide are known [6–8], but the one generally used is the synthesis shown in Scheme 1. Acetylation of (*R*)-5-bromo-3-(*N*-methylpyrrolidin-2-ylmethyl)-1*H*-indole **(9)** by using acetic anhydride and triethylamine in *N,N*-dimethylformamide affords (*R*)-1-acetyl-5-bromo-3-(*N*-methylpyrrolidin-2-ylmethyl)-1*H*-indole (**10**). When coupled in situ with phenyl vinyl sulfone (**11**) in the presence of a catalytic amount of palladium acetate [9] this affords (*R*)-1-acetyl-5-[2(phenylsulfonyl)ethyenyl]-3-(*N*-methylpyrrolidin-2-ylmethyl)-1*H*-indole (**12**), under Heck reaction conditions. Deacetylation of (**12**) by using potassium carbonate affords (*R*)-5-[(2-phenylsulfonyl)ethenyl]-3-(*N*-methylpyrrolidine-2-ylmethyl)-1*H*-indole (**13**). Reduction of **13** in the presence of palladium on carbon yields the free base of eletriptan, which on further treatment with hydrobromic acid gives eletriptan hydrobromide (**1**).

Eletriptan is used as monohydrobromide salt and has molecular formula C_22_H_27_BrN_2_O_2_S and molecular weight 463.43 amu. The maximum daily dose of eletriptan hydrobromide is 97 mg per day, which is equivalent to 80 mg of eletriptan base [10]. During the manufacture of eletriptan hydrobromide (**1**), many process-related impurities have been identified. As per ICH guide lines the acceptable level for known and unknown related impurities in the drug substance should be not more than 0.15% and 0.10%, respectively [11], depending on the maximum daily dose. To meet the meticulous regulatory requirements, the impurities present in the drug substance must be identified and characterized. By knowing the chemical structure of these impurities, control may be possible. The related compounds (impurities) of eletriptan hydrobromide were synthesized and characterized by using various spectroscopic techniques, and further confirmed by co-injection in HPLC.

## Results and Discussion

**Scheme 2:**
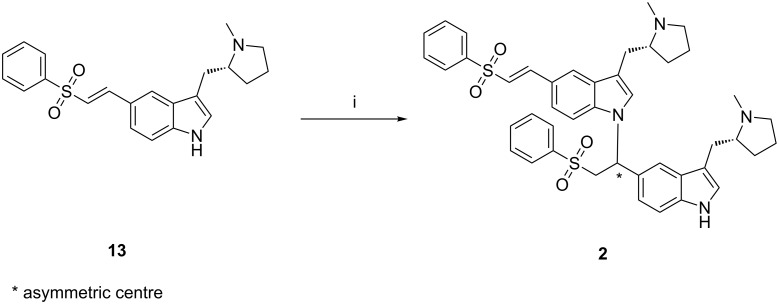
Dimerization of **13**. Reagents and conditions: (i) NaH, THF, 30–35 °C, 2 h; 11.6% yield after column chromatography.

The eletriptan dimer impurity **2** is observed at 0.3–0.5% during the basic hydrolysis of enesulfone derivative **12** and the impurity level is reduced to less than 0.20% during the isolation and purification process [12]. It is necessary to remove the impurity at this stage or control the formation during basic hydrolysis. Otherwise, the reduction rate of this impurity in later stages is low. Impurity **2** was prepared by the dimerization of desacetyl-ensulfone derivative **13** using a strong base, such as sodium hydride, under Michael addition reaction conditions in an anhydrous medium (Scheme 2). This impurity can be controlled by using hydrous conditions during the deacetylation reaction.

**Scheme 3:**
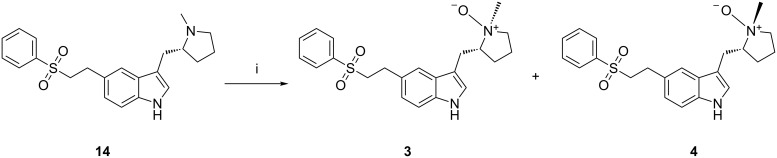
Formation of the isomers **3** and **4**. Reagents and conditions: (i) Aqueous hydrogen peroxide, methanol, ammonium molybdate, 25–30 °C, ethyl acetate; 71% yield.

Eletriptan *N*-oxide isomers **3** and **4** are possible contaminants that can be formed by oxidation in air. These compounds were prepared by oxidation of eletriptan (**14**) with aqueous hydrogen peroxide (~50%, w/w) in the presence of catalytic amounts of ammonium molybdate. The isomers **3** and **4** were separated by preparative HPLC and confirmed by ^1^H NMR spectroscopy [13–14] (Scheme 3). The combined contamination by **3** and **4** was 0.05–0.25% in eletriptan hydrobromide. Formation of this impurity can be controlled by using peroxide-free solvents during the final stage of synthesis.

**Scheme 4:**
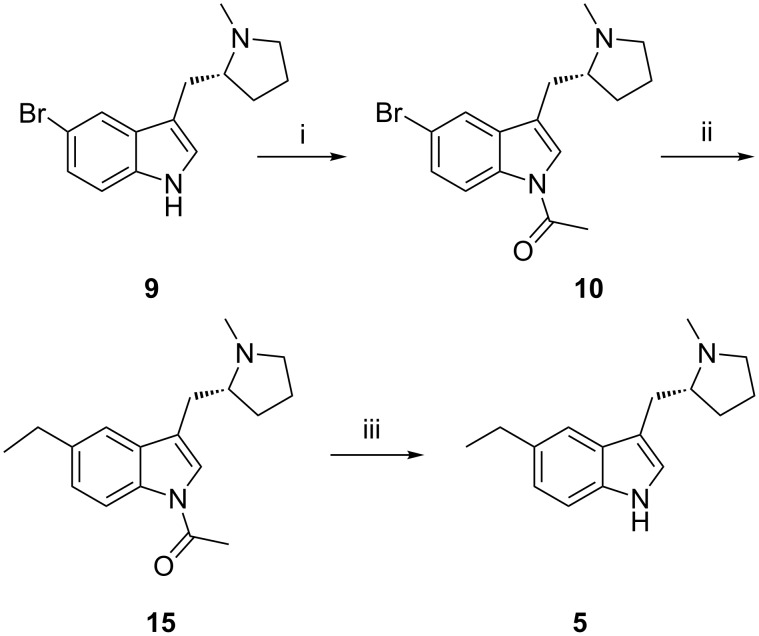
Synthesis of impurity **5**. Reagents and conditions: (i) Acetic anhydride, TEA, DMF, 90–100 °C; (ii) palladium acetate, tri-(*o*-tolyl)phosphine, K_2_CO_3_, triethyl borane in THF (1 M), THF, 64–66 °C; (iii) methanol, K_2_CO_3_, methylene chloride, H_2_O, 5–10 °C and 85% yield.

Impurity **5** is formed at 1.0–1.5% during hydrogenation due to hydrolytic cleavage. This impurity is reduced down to 0.25–0.50% during isolation and in further stages. Impurity **5** was prepared by alkylation of *N*-acetyl bromoindolyl pyrrolidine **10** with triethylborane [15–17] in the presence of a catalytic amount of palladium acetate in tetrahydrofuran, as in the following reaction scheme (Scheme 4).

**Scheme 5:**
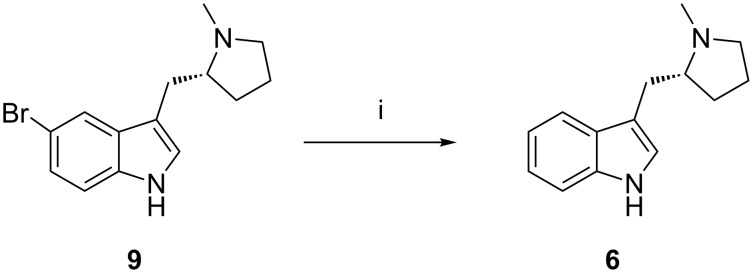
Debromination of **9** to **6**. Reagents and conditions: Palladium on carbon, ethanol, ca. 7 bar, 25–30 °C, ethyl acetate; 94.5% yield.

The content of indole pyrrolidine **6** in bromoindolyl pyrrolidine **9** is controlled by its specification to not more than 0.5%. However, during basic hydrolysis of enesulfone derivative **12**, unreacted *N*-acetyl bromoindolyl pyrrolidine **10** is converted to bromoindolyl pyrrolidine **9**. Further, **9** will convert into **6** due to debromination during the hydrogenation reaction of **13** and it can carry forward up to eletriptan hydrobromide (**1**). Indolyl pyrrolidine **6** was prepared by catalytic hydrogenation of bromoindolyl pyrrolidine (**9**) with palladium on carbon (Scheme 5). The contamination of this impurity in eletriptan hydrobromide (**1**) was 0.10–0.20%. This impurity can be controlled by tightening up the in-process control of the *N*-acetyl bromoindole pyrrolidine during the Heck reaction.

**Scheme 6:**
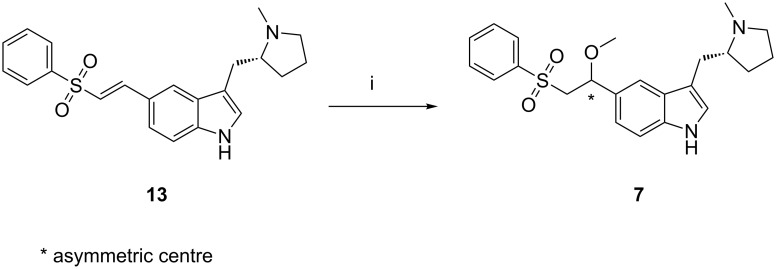
Formation of **7**. Reagents, conditions: (i) (a) K_2_CO_3_, methanol, reflux, 2 days; 40% yield after column chromatography.

During the initial process development of eletriptan, the deacetylation of enesulfone derivative **12** was performed in anhydrous methanol at ambient temperature with potassium carbonate. The reaction was completed within 30 min, but the formation of eletriptan methoxy impurity **7** was high (0.20–0.70%). Moreover, after isolation and the subsequent stage, it was still about 0.25%. To control the formation of **7**, the deacetylation reaction should be carried out in aqueous methanol instead of anhydrous methanol. Thus, a content of **7** below 0.05% is observed. Compound **7** was prepared by treating desacetyl-ensulfone **13** with potassium carbonate in anhydrous methanol (Scheme 6). This impurity can be controlled by using the hydrous conditions during the deacetylation reaction.

**Scheme 7:**
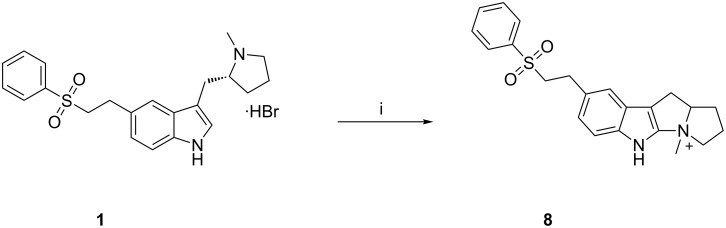
Synthesis of the tetracyclic compound **7**. Reagents and conditions: (i) aqueous hydrogen peroxide, water, acetonitrile, 80–85 °C, methylene chloride; 60% yield.

Surprisingly, we found that one unknown impurity (40–60%, by HPLC area normalization) was formed during the force degradation study of eletriptan hydrobromide (**1**) at higher temperatures (80–85 °C) in the presence of peroxide in aqueous acetonitrile. It was also observed that only 4.5% of this impurity was formed when 10% (w/w) hydrogen peroxide was used. However, this impurity was formed at 40–60%, when 30% (w/w) hydrogen peroxide used. This impurity was identified by LC–MS and characterized by ^1^H NMR, ^13^C NMR, LC–MS and FTIR. Based on the spectral data, the impurity was named as, 4-methyl-8-[2-(phenylsulfonyl)ethyl]-1,2,3,5,10,10a-hexahydropyrrolizino[3,2-*b*]indole-4-ium (tetracyclic eletriptan impurity, **8**). A similar tetracyclic impurity (UK-373,236) is also reported in literature [18]. This impurity is forming only in aprotic solvents, e.g., acetonitrile in the presence of peroxides at higher temperature. It was prepared by treating eletriptan hydrobromide (**1**) with aqueous hydrogen peroxide (30% w/w) in aqueous acetonitrile at 80–85 °C (Scheme 7). Formation of this impurity can be controlled by using peroxide-free aprotic solvents at lower temperature.

## Conclusion

We have demonstrated the synthesis and complete characterization of some of the critical impurities of eletriptan hydrobromide (**1**). This investigation helped us to establish the impurity profile of **1**.

## Supporting Information

Experimental data, IR, ^1^H NMR and ^13^C NMR, LC-MS, HPLC chromatograms for compounds **2** to **8** and HPLC chromatogram of eletriptan spiked with related compounds of eletriptan are included in the supporting information files.

File 1 Experimental data of compounds **2** to **8**.

File 2 Characterization data of compounds **2** to **8**.
