# Supplementary material for: Synthesis of compounds related to the anti-migraine drug eletriptan hydrobromide
Source: Beilstein J Org Chem. 2012 Aug 30;8:1400–5. doi: 10.3762/bjoc.8.162 (PMC3458767; doi:10.3762/bjoc.8.162)
Supplement: File 1 — Experimental data of compounds 2 to 8. [file Beilstein_J_Org_Chem-08-1400-s001.pdf]

Supporting Information

for

Synthesis of compounds related to the anti-migraine drug eletriptan hydrobromide

Suri Babu Madasu[1,2], Nagaji Ambabhai Vekariya[*1], M. N. V. D. Hari Kiran[1], Badarinadh Gupta[1], Aminul Islam[1], Paul S. Douglas[2] and Korupolu Raghu Babu[2]

Address: [1]Chemical Research and Development, Aurobindo Pharma Ltd., Survey No. 71 & 72, Indrakaran (V), Sangareddy (M), Medak Dist-502329, Andhra Pradesh, India and [2]Engineering Chemistry Department, AU College of Engineering, Andhra University, Visakhapatnam-530003, Andhra Pradesh, India

Email: Nagaji Ambabhai Vekariya – navekariya1@rediffmail.com

* Corresponding author

Experimental data of compounds 2 to 8

General

^{1}H NMR, ^{13}C NMR and DEPT spectral data were performed on a 500 and 300 MHz spectrometer in dimethyl sulfoxide (DMSO-d_6) and chloroform ($CDCl_3$). The chemical shift values were reported on the δ scale in parts per million (ppm), downfield from tetramethyl silane (TMS, δ = 0.0) as an internal standard. Spin multiplicities are given as s (singlet), d (doublet), dd (doublet of doublet), t (triplet) and m (multiplet) as well as brs (broad). Coupling constants (*J*) are given in hertz. IR spectra were recorded in the solid state as KBr dispersion by using a Perkin-Elmer "spectrum one" FTIR spectrophotometer. Mass spectra were recorded by using a Perkin-Elmer PE SCIEX-API 2000, equipped with ESI source used online with a HPLC system behind the ultraviolet (UV) detector. Chromatographic purity was determined by using the area normalization method. The solvents and reagents were used without purification.

3-[[(*R*)-1-Methylpyrrolidin-2-yl]methyl]-5-[1-[3-(((*R*)-1-methylpyrrolidin-2-yl)methyl)-5-[(*E*)-2-(phenylsulfonyl)vinyl]-1*H*-indole-1-yl]-2-[phenylsulfonyl]ethyl]-1*H*-indole (2)

Sodium hydride (21.06 g; 0.5625 mol; 60% w/w) was added to a solution of desacetyl enesulfone derivative **13** (50 g, 0.1315 mol) in tetrahydrofuran (500 mL) at 23–37 °C. The reaction mass was stirred at 30–35 °C for 2 h. DI water (300 mL) was added to the reaction mass, the product was extracted with methylene chloride (500 mL), and the solvent was evaporated up to residue. Unreacted desacetyl enesulfone derivative **13** was precipitated by adding aqueous acetonitrile (60% v/v; 400 mL) at −5 to 0 °C in the residue. Title compound was isolated from the mother liquor by extracting with methylene chloride followed by evaporation. The obtained crude material was further purified by column chromatography to give compound **2** as a brown crystalline solid (5.8 g, 11.6% yield); HPLC: 98.46%; IR (KBr, cm^{-1}): 3391 (NH), 3056 (Ar-H), 2923 (aliphatic C-H stretch), 1446 (aliphatic C-H bend), 1305 (asymmetric So_2), 1290 (C-N), 1143 (symmetric So_2), 752, 714 (Ar-H out of plane bend); ^{1}H NMR (DMSO-d_6, 300 MHz) δ 1.33 & 1.61 (2m, 4H), 1.53 (m, 4H), 2.05 & 2.92 (2m, 4H), 2.18 (m, 2H), 2.33, 2.40

& 2.89 (m, 4H), 2.25, 2.27 & 2.31 (3s, 6H), 4.19 & 5.10 (2dd, 2H), 6.14 (m, 1H), 7.09 & 7.94 (m, 20H), 10.78 (brs, 1H); ^{13}C NMR and DEPT (DMSO-d_6, 75 MHz) δ 21.9, 22.09 (CH_2), 29.3, 31.16, 31.23, 57.29 (CH_2), 58.98 (CH_2), 65.91, 66.14, 66.48, 66.67 (CH_2), 111.12, 111.67 (CH), 112.92 (C), 114.79 (C), 116.90, 116.99 (CH), 120.24 (CH_2), 121.97, 122.08 (CH), 123.70 (C), 124.10, 124.18 (CH), 125.05, 125.18 (CH), 127.34, 127.61 (CH), 127.93 (C), 128.63, 128.82 (C), 129.04 (CH), 129.35 (C), 129.98 (CH), 133.66 (CH), 135.94 (C), 137.46 (C), 139.40, 139.44 (C), 142.06 (C), 144.58 (CH), 144.61 (C); MS *m/z* (ESI): 761.3 $[M + H]^+$, 759.6 $[M - H]^-$.

(*R*)-3-[(1-Methyl-2-pyrrolidinyl)methyl]-5-[2-(phenylsulfonyl)ethyl]-*N*-oxide-1*H*-indole (3)

Aqueous hydrogen peroxide (26.5 g; 0.389 mol; 50% w/w) was added to a solution of eletriptan free base **14** (50 g; 0.131 mol) in methanol (500 mL) at 25–30 °C. To the resulting mixture, a catalytic amount ammonium molybdate (0.1 g) was added, and the mixture was kept under stirring for about 15 h at ambient temperature. After completion of the reaction, the reaction mass was evaporated, and the compound precipitated after the addition of ethyl acetate (250 mL). The resulting suspension was filtered, washed with ethyl acetate and dried to obtain a mixture of eletriptan *N*-oxide isomers **3** and **4**. Further separation by preparative HPLC yielded the pure eletriptan *N*-oxide-1 **3** (9.25 g, 17.75% yield); HPLC: 95.17%; IR (KBr, cm^{-1}): 3391 (NH), 2923 (aliphatic C-H stretch), 1446 (aliphatic C-H bend), 1233 (C-N), 761, 739 (Ar-H out of plane bend); ^{1}H NMR ($CDCl_3$, 500 MHz) δ 1.62 & 2.10 (2m, 2H), 1.79 & 1.97 (2 m, 2H), 2.60 & 3.53 (2m, 2H), 3.06 (t, 2H), 3.12 (s, 3H), 3.38 (m, 2H), 3.58 (t, 2H), 3.79 (m, 1H), 6.83 (d, 1H), 6.98 (s, 1H), 7.29 (d, *J* = 8.0 Hz, 1H), 7.34 (s, 1H), 7.52 (dd, *J* = 9.0 Hz, 2H), 7.61 (dd, *J* = 8.0 Hz, 1H), 7.90 (d, *J* = 8.0 Hz, 2H); ^{13}C NMR and DEPT ($CDCl_3$, 75 MHz) δ 18.78 (CH_2), 24.91 (CH_2), 27.31 (CH_2), 28.90 (CH_2), 49.66 (CH_3), 58.19 (CH_2), 70.22 (CH_2), 79.52 (CH), 109.1 (C), 112.1 (CH), 117.6 (CH), 122.2 (CH), 123.9 (CH), 127.3 (C), 128.01 (CH,CH), 128.05 (CH, CH), 129.3 (CH), 133.7 (C), 135.5 (C), 139.0 (C); MS *m/z* (ESI): 399.3 $[M + H]^+$.

(*R*)-3-[(1-Methyl-2-pyrrolidinyl)methyl]-5-[2-(phenylsulfonyl)ethyl]-*N*-oxide-1*H*-indole (4)

As obtained from the procedure above, the mixture of eletriptan *N*-oxide isomers **3** and **4** was separated by preparative HPLC to get the pure eletriptan *N*-oxide-2 **4** (27.75 g, 53.26% yield); HPLC: 94.62%; IR (KBr, cm^{-1}): 3400 (NH), 2957 (aliphatic C-H stretch), 1446 (aliphatic C-H bend), 1232 (C-N), 760, 731 (Ar-H out of plane bend); ^{1}H NMR ($CDCl_3$, 500 MHz) δ 1.84 & 2.34 (2m, 2H), 1.97 & 2.16 (2m, 2H), 3.15 (m, 2H), 3.22 & 3.41 (2m, 2H), 3.29 (s, 3H), 3.39 (m, 2H), 3.45 & 3.87 (2t, 2H), 3.61 (m, 1H), 6.93 (d, *J* = 8.5 Hz, 1H), 7.09 (s, 1H), 7.30 (d, *J* = 9.0 Hz, 1H), 7.31 (s, 1H), 7.58 (dd, *J* = 8.0 Hz, 2H), 7.67 (dd, *J* = 8.0 Hz), 7.95 (d, *J* = 8.0 Hz, 1H), 8.93 (S, 1H);^{13}C NMR and DEPT ($CDCl_3$, 500 MHz) δ 19.49 (CH_2), 23.76 (CH_2), 28.77 (CH_2), 28.89 (CH_2), 54.04 (CH_3), 58.34 (CH_2), 70.61 (CH_2), 76.75 (CH), 110.7 (C), 111.9 (CH), 117.8 (CH), 122.5 (CH), 123.8 (CH), 127.4 (C),128.0 (CH, CH), 128.4 (C), 129.3 (CH, CH), 133.7 (CH), 133.5 (C), 139.2 (C); MS *m/z* (ESI): 399.0 $[M + H]^+$.

(*R*)-3-[(1-Methyl-2-pyrrolidinyl)-methyl]-5-ethyl-1*H*-indole (5)

A solution of (*R*)-5-bromo-3-(*N*-methylpyrrolidin-2ylmethyl)-1*H* indole **(9)** (5 g; 0.017 mol) in *N,N*-dimethylformamide (8.3 mL) containing triethylamine (2.1 g; 0.021 mol) was treated with acetic anhydride (2.1 g; 0.021 mol) over 10 min, then the mixture was treated to 95–100 °C for 4 h and allowed to cool. The resulting *N*-acetyl BIP **10** precipitated by quenching of the reaction mass into DM water, and the solid was collected by filtration then dried in vacuum (5.1 g). A solution of *N*-acetyl BIP **10** (5 g; 0.0149 mol) in tetrahydrofuran (50 mL) containing potassium carbonate (4.12 g; 0.0298 mol), palladium acetate (0.5 g; 0.0022 mol), tri-(*o*-tolyl)phosphine (2.72 g; 0.00897 mol) was treated with 1.0 M solution of triethylborane in tetrahydrofuran (44.9 mL; 0.0449 mol) at 30–35 °C, and the reaction mixture was heated under reflux for 6–7 h. The reaction was cooled to 0 °C and quenched by 10% aqueous sodium hydroxide solution. After stirring for 30 min at 25 °C, the mixture was acidified with dilute aqueous hydrochloride and extracted with methylene chloride (2 × 50 mL) and concentrated

under reduced pressure to obtained compound **15** as an oily residue. Compound **15** was dissolved in methanol (50 mL) and treated with potassium carbonate (1.03 g; 0.0075 mol) at ambient temperature for 1 h. The solvent was evaporated followed by the addition of DI water (50 mL), the product was extracted with methylene chloride (2 × 25 mL), and the solvent was evaporated to residue, which was further purified by column chromatography to give compound **5** as a brown crystalline solid (30 g; 85% yield); HPLC: 95.88%; IR (KBr, cm^{-1}): 3411 (NH), 3134 (Ar C-H), 2963 (aliphatic C-H stretch), 1454 (aliphatic C-H bend), 1224 (C-N), 762, 703 (Ar-H out of plane bend); ^{1}H NMR ($CDCl_3$, 300 MHz) δ 1.29 (t, 3H), 1.62 & 1.82 (2m, 4H), 2.23 & 2.59 (2m, 2H), 2.48 (s, 3H), 2.50 & 3.15 (2m, 2H), 2.76 (q, 2H), 317 (m, 1H), 7.00 (s, 1H), 7.05 (dd, 1H), 7.26 (d, 1H), 7.39 (d, 1H), 7.97 (brs, 1H); ^{13}C NMR and DEPT ($CDCl_3$, 75 MHz) δ 16.53 (CH_3), 21.85 (CH_2), 29.08 (CH_2), 29.91 (CH_2), 31.51 (CH_2), 40.79 (CH), 57.55 (CH_2), 66.63 (CH), 110.83 (CH), 113.78 (C), 117.30 (CH), 122.1 (CH), 122.45 (CH), 127.90 (C), 134.72 (C), 135.14 (C); MS *m/z* (ESI): 243.3 $[M + H]^+$.

(*R*)-3-(1-Methyl-2-pyrrolidinyl)methyl)-1*H*-indole (6)

5% Palladium on carbon (1.0 g; 50% wet) was added to a solution of bromoindole proline **9** (BIP; 10 g; 0.034 mol) in ethanol (120 mL). The mixture was exposed to hydrogen (7 bar) at ambient temperature for about 2 h. After completion of the reaction, we evaporated the reaction mass and precipitated the product by adding ethyl acetate (70 mL). The resulting suspension was filtered, washed with ethyl acetate and dried to obtain the title compound **6** (6.9 g; 94.5% yield); HPLC: 100.00%; IR (KBr, cm^{-1}): 3428 (NH), 3102, 3054 (Ar-H), 2972, 2947 (aliphatic C-H stretch), 1451 (aliphatic C-H bend), 1223 (C-N), 765, 739 (Ar-H out of plane bend); ^{1}H NMR ($CDCl_3$, 300 MHz) δ 1.63 & 1.82 (2m, 4H), 2.27 & 2.68 (2m, 2H), 2.48 (s, 3H), 2.57 & 3.20 (2m, 2H), 3.23 (m, 1H), 7.03 (s, 1H), 7.11 & 7.18 (2t, 2H), 7.35 & 7.60 (2d, 2H), 8.24 (brs, 1H); ^{13}C NMR and DEPT ($CDCl_3$ 75 MHz) δ 21.8 (CH_2), 29.9 (CH_2), 31.5 (CH_2), 40.7 (CH_3),

57.5 (CH_2), 66.6 (CH), 111.1 (CH), 113.9 (C), 118.8 (CH), 119.0 (CH), 121.7 (CH), 121.9 (CH), 127.6 (C), 136.2 (C); MS *m/z* (ESI): 214.9 $[M + H]^+$.

(*R*)-3-[(1-Methyl-2-pyrrolidinyl)methyl]-5-[2-(phenylsulfonyl)-1-methoxyethyl]-1*H*-indole (7)

Desacetyl enesulfone derivative **13** (10 g; 0.0263 mol) was dissolved in methanol (150 mL) at 25–30 °C, and potassium carbonate (14.5 g; 0.1050 mol) was added. The resulting mixture was heated under reflux for 2 days, allowed to cool to room temperature then evaporated under reduced pressure. From the obtained foam material, inorganic salts were removed by adding methylene chloride (50 mL) and DI water (20 mL). The desired compound **7** was extracted from methylene chloride by evaporation to obtain an oil and thereafter purified by column chromatography (ethyl acetate/methanol as eluent) to provide the title compound **7** (4.3 g; 40% yield); HPLC: 97.66%: IR (KBr, cm^{-1}): 3379 (NH), 3037 (Ar-H), 2938 (aliphatic C-H), 1447 (C-H bend), 1213 (C-H stretch), 789, 748 (Ar-H out of plane bend); ^1H NMR (DMSO-d_6, 300 MHz) δ 1.46 & 1.68 (2m, 2H), 1.57 (m, 2H), 2.13 & 2.98 (2m, 2H), 2.35 (s, 3H), 2.38 (m, 1H), 2.40 & 2.99 (2m, 2H), 2.91 (s, 3H), 3.49 & 4.64 (2m, 2H), 3.97 (m, 1H), 6.96 (d, 1H), 7.14 (s, 1H), 7.27 (d, 1H), 7.40 (s, 1H), 7.59 (dd, 2H), 7.69 (dd, 1H), 7.90 (d, 2H), 10.82 (s, H); ^{13}C NMR and DEPT (DMSO-d_6, 75 MHz) δ 21.54 (CH_2), 29.06 (CH_2), 30.83 (CH_2), 41.30 (CH_3), 56.18 (OCH_3), 56.84 (CH_2), 62.19 (CH_2), 66.94 (CH), 79.46 (CH), 112.34 (CH), 112.43 (C), 118.08 (CH), 120.43 (CH), 124.4 (CH), 127.32 (C), 128.19 (CH), 128.22 (C), 128.60 (CH), 129.77 (CH), 134.20 (CH), 136.0 (CH); MS *m/z* (ESI): 413.3 $[M + H]^+$, 411.3 $[M - H]^-$.

4-Methyl-8-[2-(phenylsulfonyl)ethyl]-1,2,3,5,10,10a-hexahydropyrrolidino[3,2-b]indol-4-ium (8)

Aqueous hydrogen peroxide solution (80 mL, 30% w/w; 0.8333 mol) was added to a solution of eletriptan hydrobromide (**1**, 0.5 g; 0.001 mol) in aqueous acetonitrile (500 mL; prepared by mixing 375 mL water with 125 mL acetonitrile) at 20–25 °C. The resulting solution was heated to 80–85 °C for about 45 min. Thereafter, the contents were allowed to cool to room temperature. The product was extracted

with methylene chloride and washed with water. Evaporation of the organic layer yielded an oily residue. Thereafter, the desired compound **8** was purified by flash chromatography (ethyl acetate/methanol as eluent) to give solid (0.3 g, 60% yield); HPLC: 92.40%; IR (KBr, cm^{-1}): 3436 (NH), 3062 (Ar-H), 2926 (aliphatic C-H), 1446 (C-H bend), 1226 (CN stretch), 737 (Ar-H out of plane); ^{1}H NMR (DMSO-d_6, 500 MHz) δ 1.83 & 2.15 (2m, 2H), 1.93 & 2.48 (2m, 2H), 2.78 & 3.4 (2m, 2H), 2.86 (m, 2H), 3.51 (m, 2H), 3.67 & 3.91 (2m, 2H), 4.90 (m, 1H), 6.63 (d, 1H), 7.02 (s, 1H), 7.18 (d, 1H), 7.65 (dd, 2H), 7.78 (dd, 1H), 7.96 (d, 2H); ^{13}C NMR and DEPT (DMSO-d_6, 125 MHz) δ 25.25 (CH_2), 29.16 (CH_2), 29.72 (CH_2), 32.86 (CH_2), 53.13 (CH_3), 57.35 (CH_2), 65.41 (CH_2), 87.54 (CH), 100. 16 (C), 11.40 (CH), 118.84 (CH), 124.81 (CH), 125.16 (C), 128.05 (CH), 129.88 (CH), 134.36 (CH), 139.09 (C), 147.70 (C), 152.84 (C); MS *m*/*z* (ESI): 381.0 $[M + H]^+$.
