# Supplementary material for: Synthesis of compounds related to the anti-migraine drug eletriptan hydrobromide
Source: Beilstein J Org Chem. 2012 Aug 30;8:1400–5. doi: 10.3762/bjoc.8.162 (PMC3458767; doi:10.3762/bjoc.8.162)
Supplement: File 2 — Characterization data of compounds 2 to 8. [file Beilstein_J_Org_Chem-08-1400-s002.pdf]

Supporting Information

for

Synthesis of compounds related to the anti-migraine drug eletriptan hydrobromide

Suri Babu Madasu[1,2], Nagaji Ambabhai Vekariya[*1], M. N. V. D. Hari Kiran[1], Badarinadh Gupta[1], Aminul Islam[1], Paul S. Douglas[2] and Korupolu Raghu Babu[2]

Address: [1]Chemical Research and Development, Aurobindo Pharma Ltd., Survey No. 71 & 72, Indrakaran (V), Sangareddy (M), Medak Dist-502329, Andhra Pradesh, India and [2]Engineering Chemistry Department, AU College of Engineering, Andhra University, Visakhapatnam-530003, Andhra Pradesh, India

Email: Nagaji Ambabhai Vekariya - navekariya1@rediffmail.com

* Corresponding author

Characterization data of compounds 2 to 8

ELETRIPTAN DIMER CHP (1641) 160 A.R.NO: S-01011

DMSO-d6

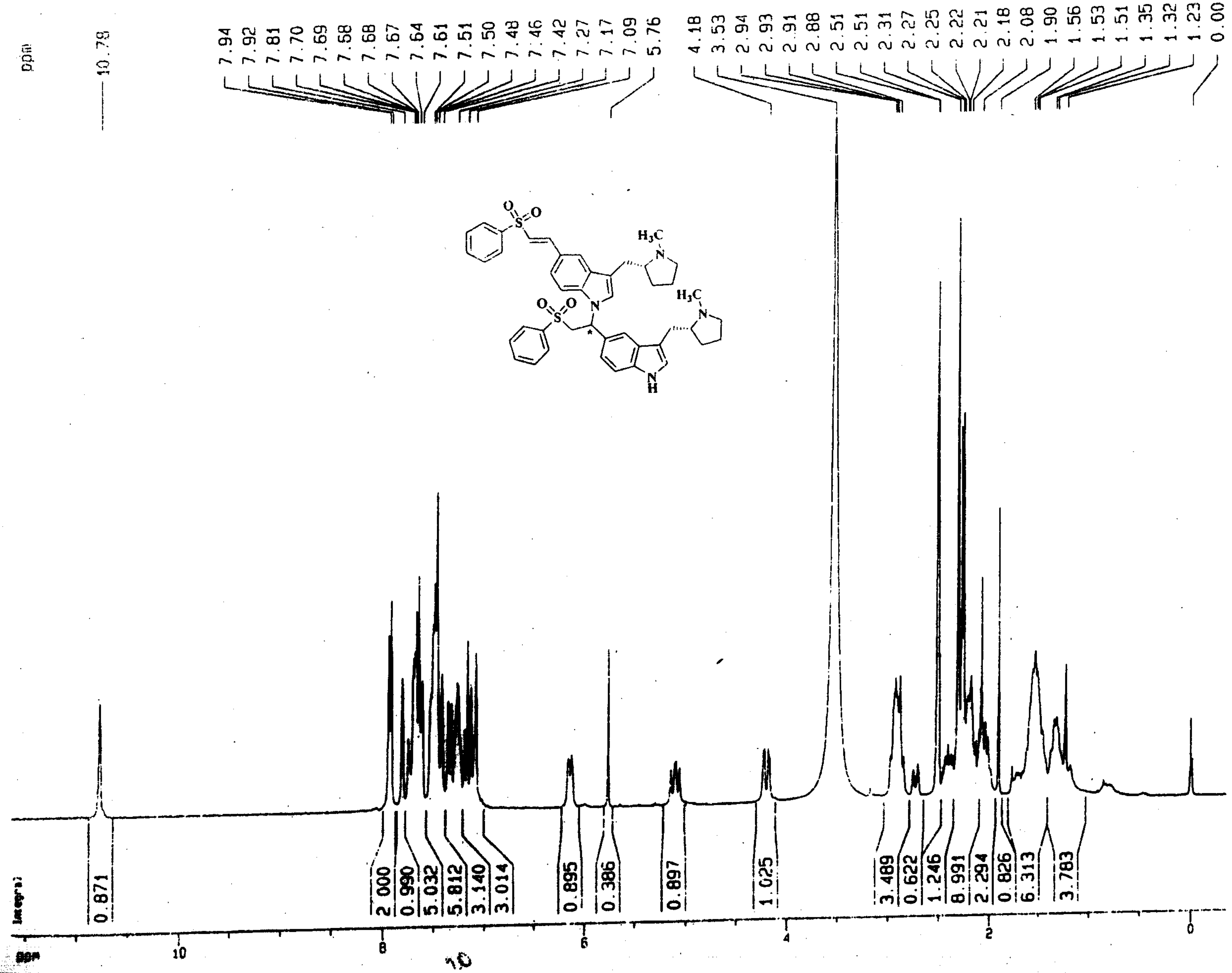

APL-RESEARCH CENTRE

Current Data Parameters

NAME	cnp-1641-160
EXPNO	1
PROCNO	1

F2 - Acquisition Parameters

Date_	20101101
Time	9.35
INSTRUM	dpx300
PROBHD	5 mm BBO BB-
PULPROG	zg30
TD	32768
RG	203.[illegible]
SOLVENT	DMSO
NS	16
AQ	1.8219508 sec
DW	55.600 usec
DE	6.00 usec
D1	2.00000000 sec
P1	6.75 usec
PL1	-6.00 dB
SFO1	300.1315006 MHz

======== CHANNEL f1 ========

NUC1	1H

F2 - Processing parameters

SI	32768
SF	300.1299993 MHz
WDW	EM
LB	0.30 Hz
GB	0

1D NMR plot parameters

CX	20.50 cm
F1P	11.634 ppm
F2P	-0.351 ppm

PENDANT-NMR of ELETRIPTAN DIMER CHP(1641) 160 A.R.NO: S-01011

DMSO-d6

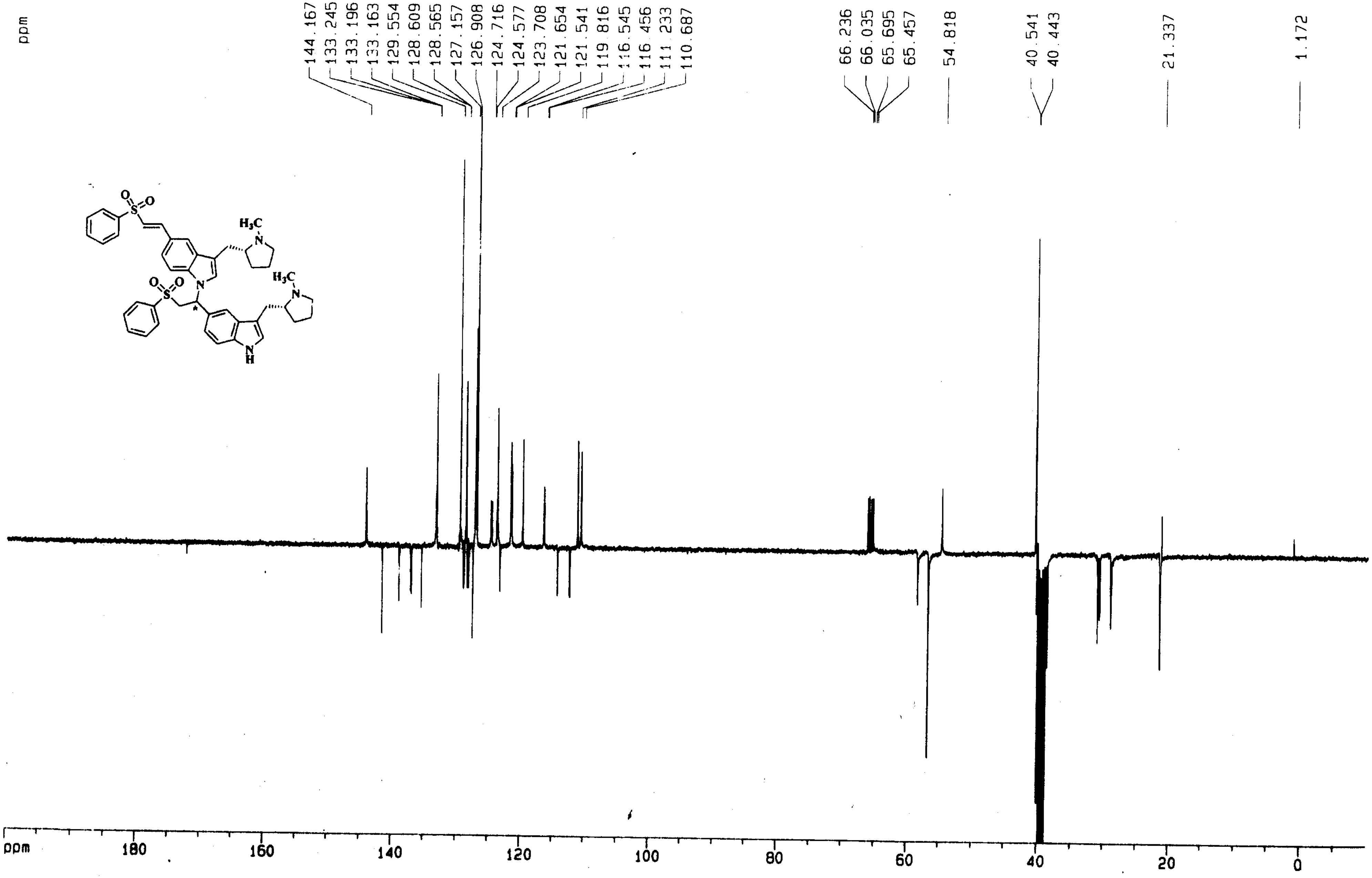

Analyst Version: 1.4.2
Acq. Time: 11:33
Acq. Date: Tuesday, November 02, 2010

APL RESEARCH CENTRE
ANALYTICAL RESEARCH DEPARTMENT

ample Comment: B.NO:CHP(1641)160
Sample Name: ELETRIPTAN DIMER
Acq. File: NOV_02_CHP(1641)160_P_F_M.wiff

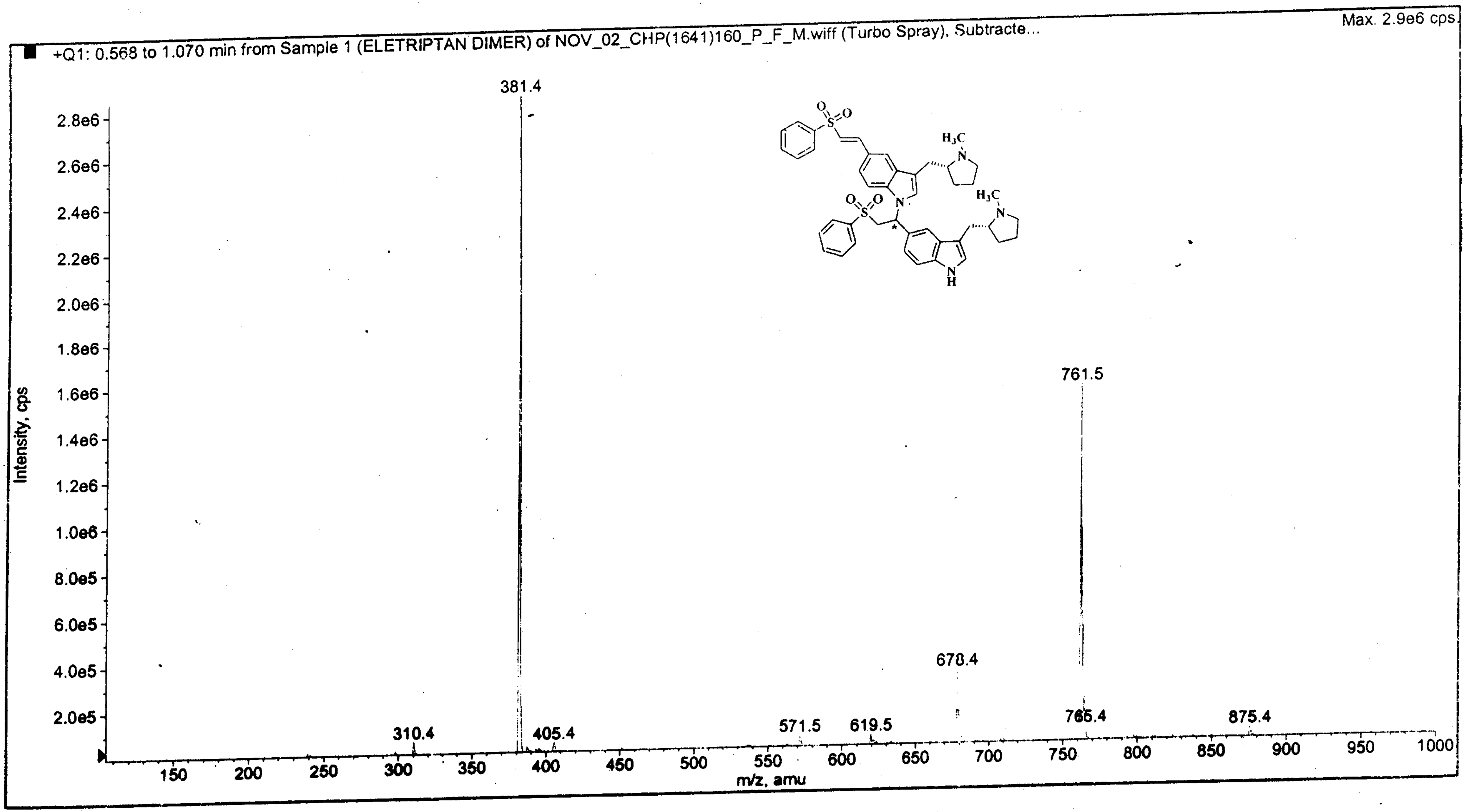

K.P.R.
Analyst

Period/Expt.: Period 1, Experiment 1
Scan Mode: Profile
Polarity/Scan Type: Positive Q1 MS

Date: Monday. May 23, 2011

APL RESEARCH CENTRE

ARE_018

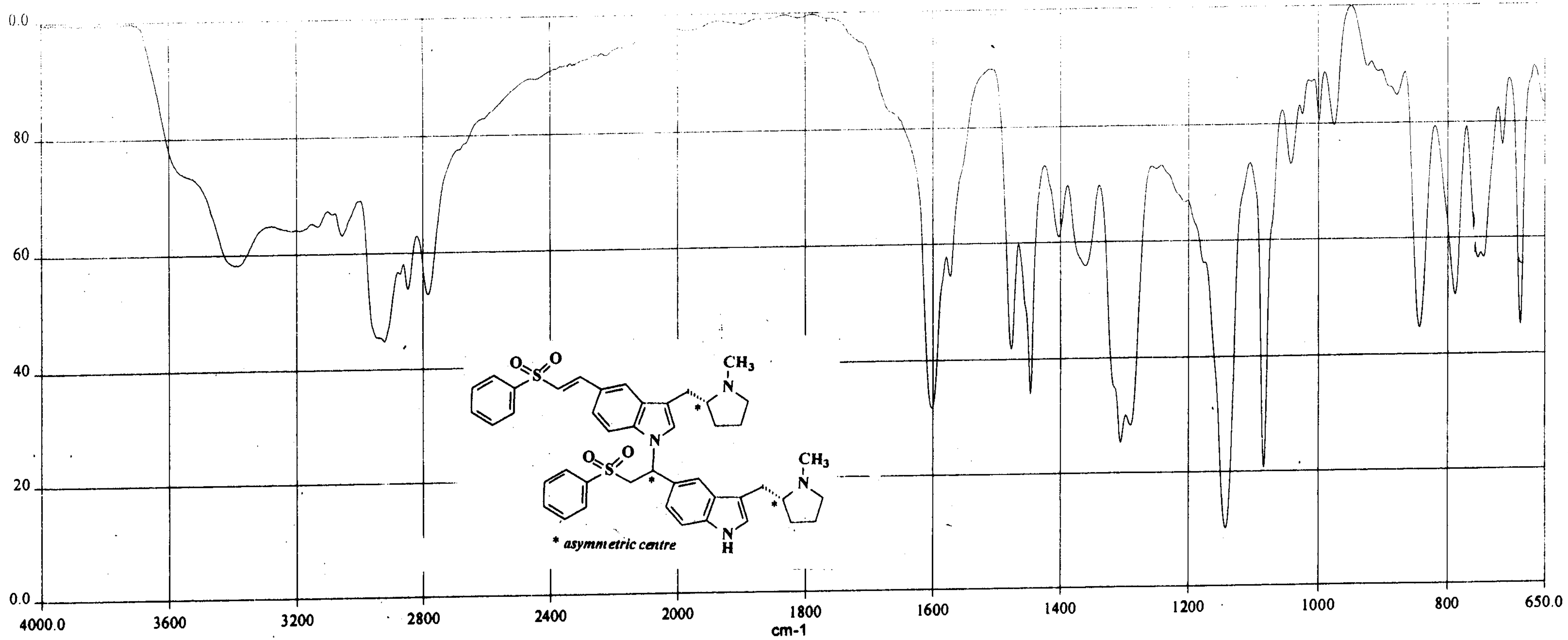

Status (ELETRIPTAN DIMER)

Filename: ELETRIPTAN DIMER-1&&2-CHP(1641)160.002
Date Created: Monday, May 23, 2011 11:03 AM India Standard Time
Analyst: Administrator
Description: B.NO.: CHP(1641)160
Comments:
SAMPLE: ELETRIPTAN HBr DIMER-1&2
PROJECT: ELETRIPTAN HBr
2.04 mg OF SAMPLE IN 427 mg OF KBr

Abscissa: (cm-1)
Start: 4000.00
End: 450.00
Interval: -1.000000

Ordinate: (%T)
Maximum: 100.00
Minimum: 10.00
Points: 3551

Instrument Model : Spectrum One
Instrument Serial Number : 53009

ELETRIPTAN DIMER-1&2-CHP(1641)160.pk

ELETRIPTAN DIMER-1&2-CHP(1641)160.002 3351 4000.00 650.00 10.00 99.86 4.00 %T 8 1.00
B.NO.: CHP(1641)160
REF 4000 99.69 2000 97.55 600
3391.40 58.16 3056.39 63.10 2923.62 44.86 2848.85 53.84 2784.98 52.83
1601.36 31.72 1572.63 54.55 1476.32 41.80 1446.57 34.00 1402.80 60.94
1360.53 55.99 1305.42 25.23 1290.33 28.24 1143.06 9.99 1084.20 20.64
1042.23 73.10 1024.40 81.67 998.88 80.51 975.69 79.73 878.36 84.78
843.40 44.68 787.94 50.23 752.60 56.43 714.54 76.06 687.29 45.03

4

23/05/2011

APL RESEARCH CENTRE
ANALYTICAL RESEARCH DEPARTMEN'

INSTRUMENT ID : RCII_AE088 PROJECT NAME : MAR_2012\RCII_AE088

Sample ID	CHP(1641)160/Eletriptan Dimer1&2 (ELETRIPTAN DIMER)	Proc. Chnl. Descr :	W2996 PDA 225.0 nm
Run Time	60.0 Minutes	Date Acquired :	3/29/2012 10:09:10 PM IST
Vial	33	Acq. Method Set :	Eletriptan_RS_MET
Injection	1	Date Processed :	3/30/2012 5:17:25 PM IST
Injection Volume	20.00 ul	Processing Method :	Eletriptan_RS_Pro

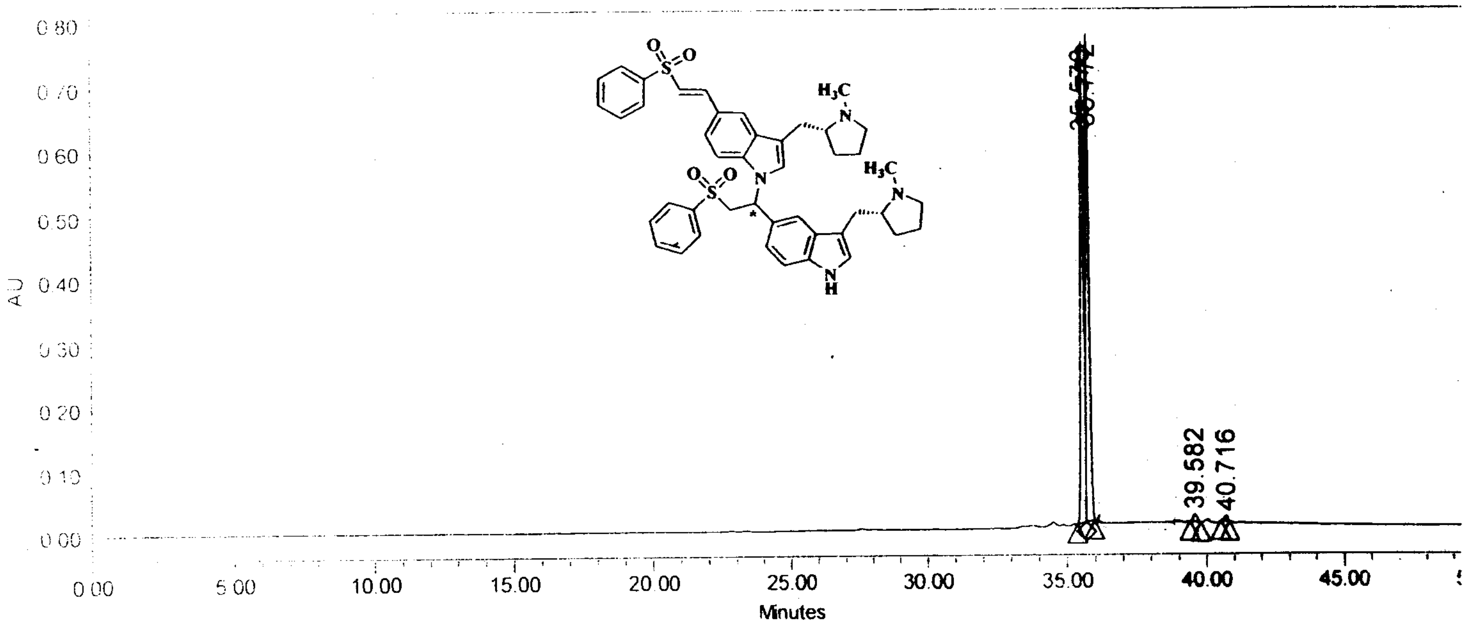

Peak Results

	RT (min)	Area (µV*sec)	% Area	RT Ratio	Name
1	35.58	5928850	47.96	1.00	Eletriptan Dimer-1
2	35.77	6243522	50.50	1.01	Eletriptan Dimer-2
3	39.58	118176	0.96	1.11	Peak9
4	40.72	72638	0.59	1.14	Peak10

ELETRIPTAN N-OXIDES-1 CHP (1708) 21 A.R.NO: S-01012671
DMSO-d6

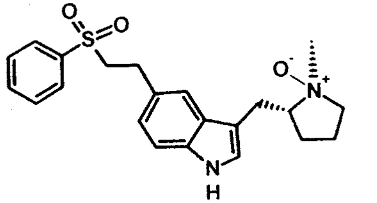

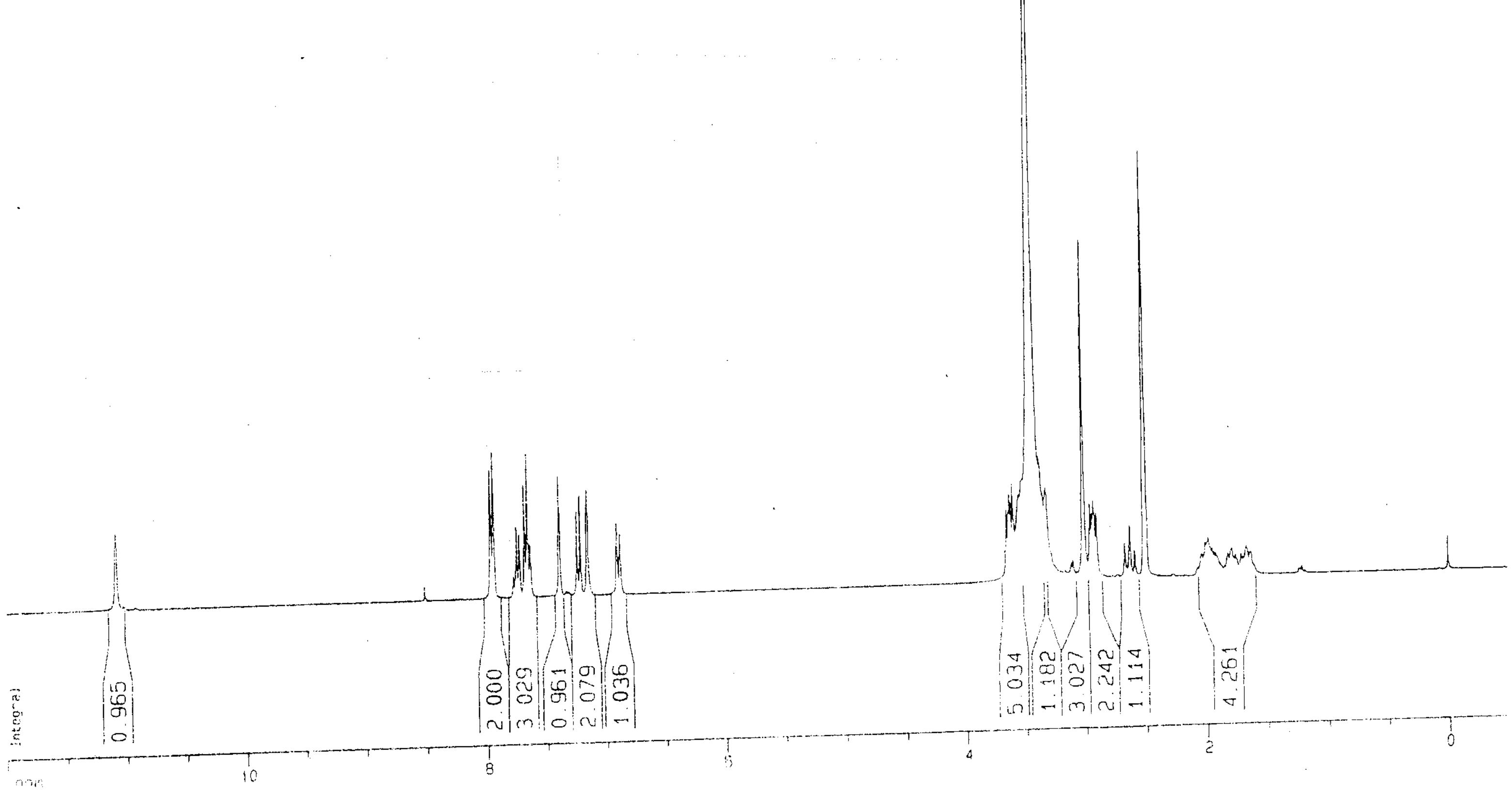

APL-RESEARCH CENTRE

Current Data Parameters
NAME chp-1708-21
EXPNO 1
PROCNO 1

F2 - Acquisition Parameters
Date_ 20101215
Time 9.56
INSTRUM dpx300
PROBHD 5 mm BBO BB-
PULPROG zg30
TD 32768
RG 256
SOLVENT DMSO
NS 113
AQ 1.8219508 sec
DW 55.600 usec
DE 6.00 usec
D1 2.00000000 sec
P1 6.75 usec
PL1 -6.00 dB
SFO1 300.1315006 MHz
============ CHANNEL f1 ============
NUC1 1H

F2 - Processing parameters
SI 32768
SF 300.1300003 MHz
WDW EM
LB 0.30 Hz
GB 0

1D NMR plot parameters
CX 20.50 cm
F1P 12.000 ppm
F2P -0.500 ppm

Gupta

SAMPLE: chp-1761-14

Eletriptan N-oxide-I
chp-1761-14

Solvent: cdcl3
Temp. 25.0 C / 298.1 K
Operator: apl
File: DEPT_01
VNMRS-500 "APLV500"

PULSE SEQUENCE: DEPT
Relax. delay 1.000 sec
Pulse 90.0 degrees
Acq. time 1.022 sec
Width 31250.0 Hz
8000 repetitions

OBSERVE C13, 125.6793948
DECOUPLE H1, 499.8207327
Power 39 dB
on during acquisition
off during delay
WALTZ-16 modulated

DATA PROCESSING
Line broadening 0.5 Hz
FT size 65536
Total time 4.5 hours

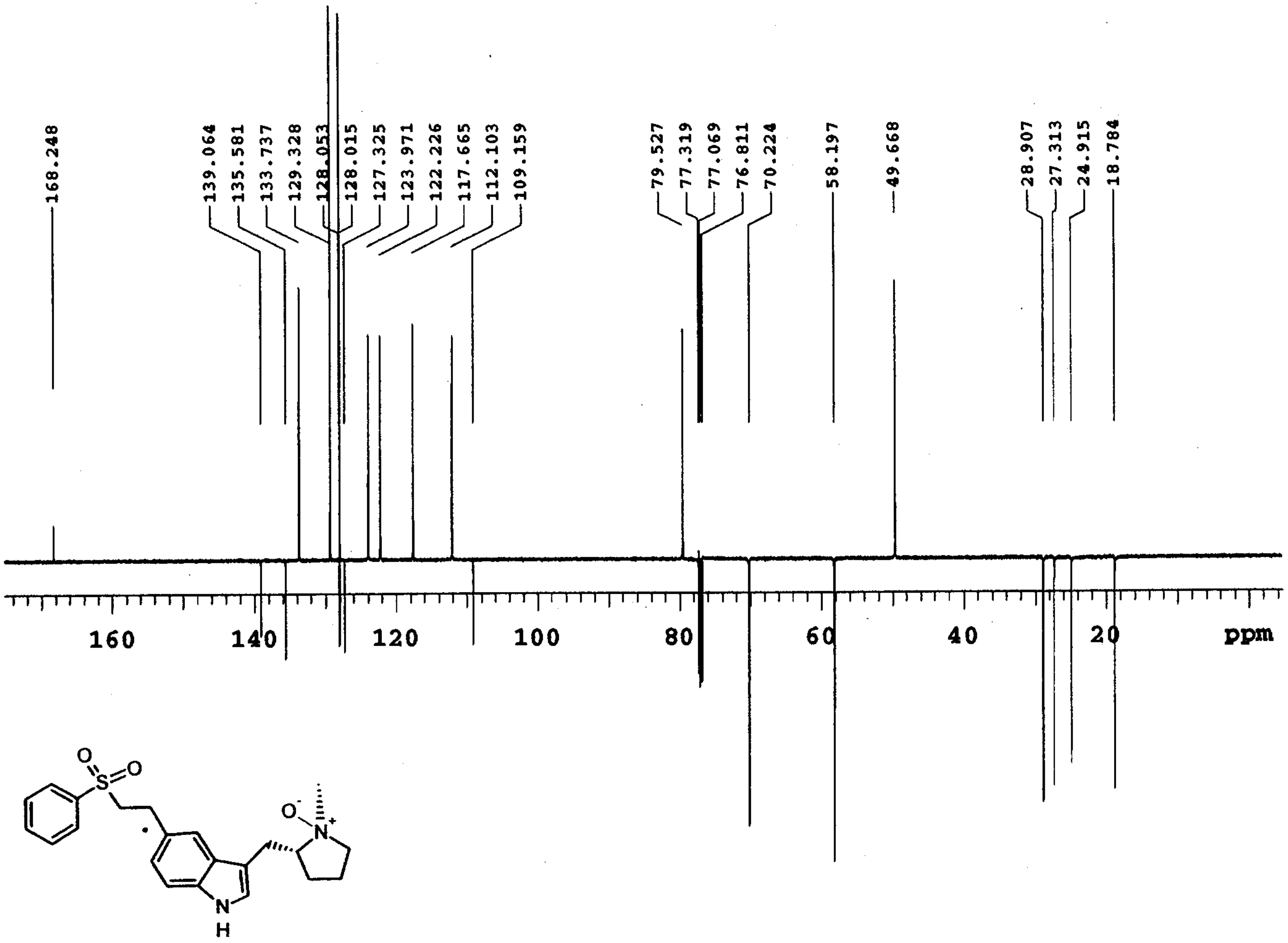

Archive directory: /home/apl/vnmrsys/data/2011/Jun | Sample: chp-1761-14_20110616 | File: DEPT_01

Analyst Version: 1.4.2
Acq. Time: 11:23
Acq. Date: Wednesday, December 15, 2010

APL RESEARCH CENTRE
ANALYTICAL RESEARCH DEPARTMENT

Sample Comment: B NO:CHP(1708)21
Sample Name: ELETRIPTAN UNK IMP RRT AT
Acq. File: DEC_15_CHP(1708)21_P_F_M.wiff

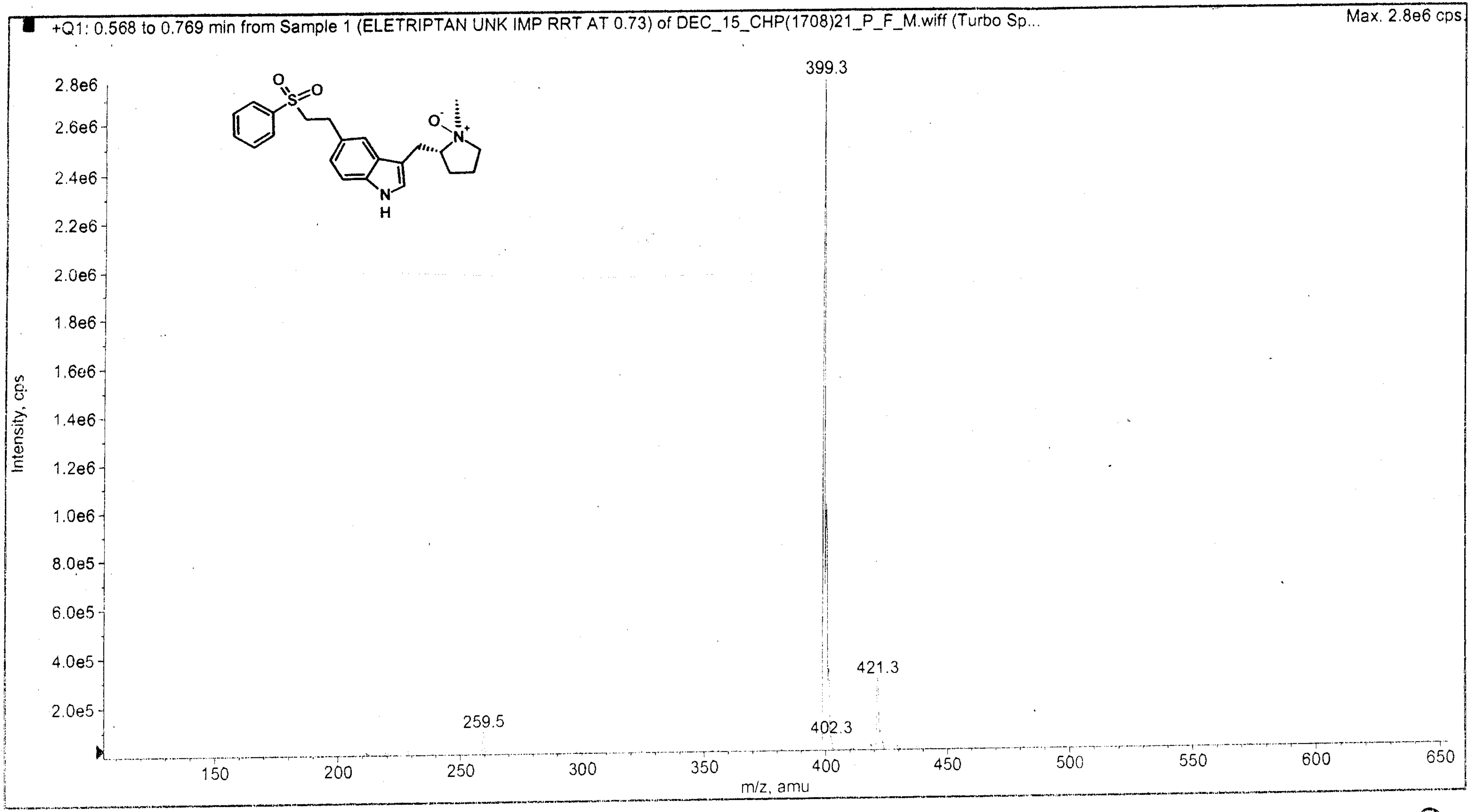

Analyst

Period/Expt.: Period 1, Experiment 1
Scan Mode: Profile
Polarity/Scan Type: Positive Q1 MS

Date: Monday, May 23, 2011

APL RESEARCH CENTRE

ARE_018

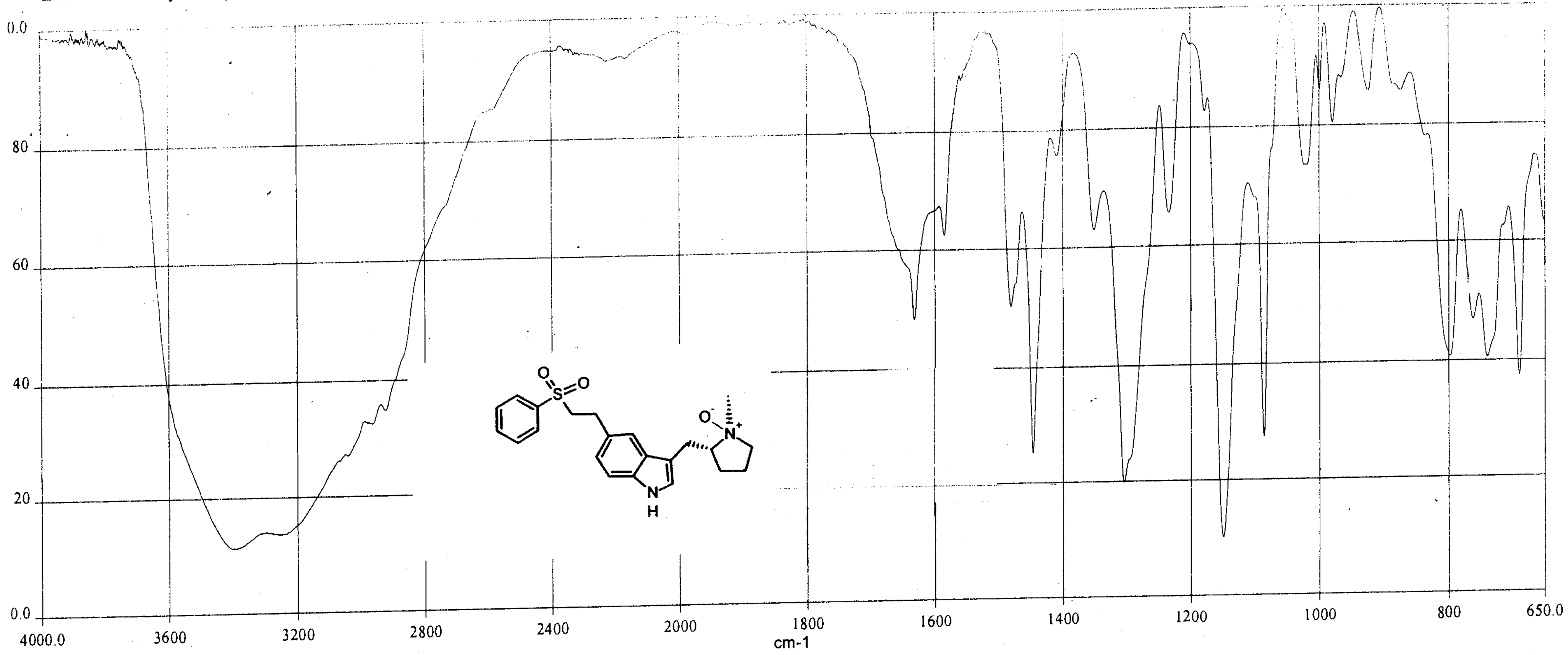

ELETRIPTAN HBr N-OXIDE-1-CHP(1708)21.pk

ELETRIPTAN HBr N-OXIDE-1-CHP(1708)21.002 3351 4000.00 650.00 10.00 100.00 4.00 %T 8 1.0(
B.NO.: CHP(1708)21
REF 4000 99.08 2000 97.49 600

3910.67	97.65	3874.71	97.59	3864.39	97.84	3846.49	97.36	3826.59	97.31
3810.96	97.20	3758.36	96.62	3391.62	11.18	2923.69	34.76	2229.39	92.87
1632.09	48.36	1584.60	62.45	1480.47	50.35	1446.29	25.08	1409.28	75.78
1350.72	63.08	1303.71	19.79	1233.41	65.71	1177.79	82.71	1148.88	9.99
1085.84	27.21	1022.81	73.43	999.03	85.91	979.31	80.47	924.64	85.70
873.89	85.73	797.32	40.76	761.80	46.85	739.45	40.53	689.55	37.28

Status

Filename: ELETRIPTAN HBr N-OXIDE-1-CHP(1708)21.002
Date Created: Monday, May 23, 2011 10:48 AM India Standard Time
Analyst: Administrator
Description: B.NO.: CHP(1708)21
Comments:
SAMPLE: ELETRIPTAN HBr N-OXIDE-1
PROJECT: ELETRIPTAN HBr
1.91 mg OF SAMPLE IN 403 mg OF KBr

Abscissa: (cm-1)
Start: 4000.00
End: 450.00
Interval: -1.000000

Ordinate: (%T)
Maximum: 100.00
Minimum: 10.00
Points: 3551

Instrument Model: Spectrum One
[illegible]

23/05/2011

APL RESEARCH CENTRE
ANALYTICAL RESEARCH DEPARTMEN

INSTRUMENT ID : RCII_AE088

PROJECT NAME : MAR_2012\RCII_AE088

Sample ID	CHP(1708)21/Eletriptan -N-Oxide-1	Proc. Chnl. Descr :	W2996 PDA 225.0 nm
Run Time	60.0 Minutes	Date Acquired :	3/29/2012 8:07:02 PM IST
Vial	31	Acq. Method Set :	Eletriptan_RS_MET
Injection	1	Date Processed :	3/30/2012 5:16:09 PM IST
Injection Volume :	20.00 ul	Processing Method :	Eletriptan_RS_Pro

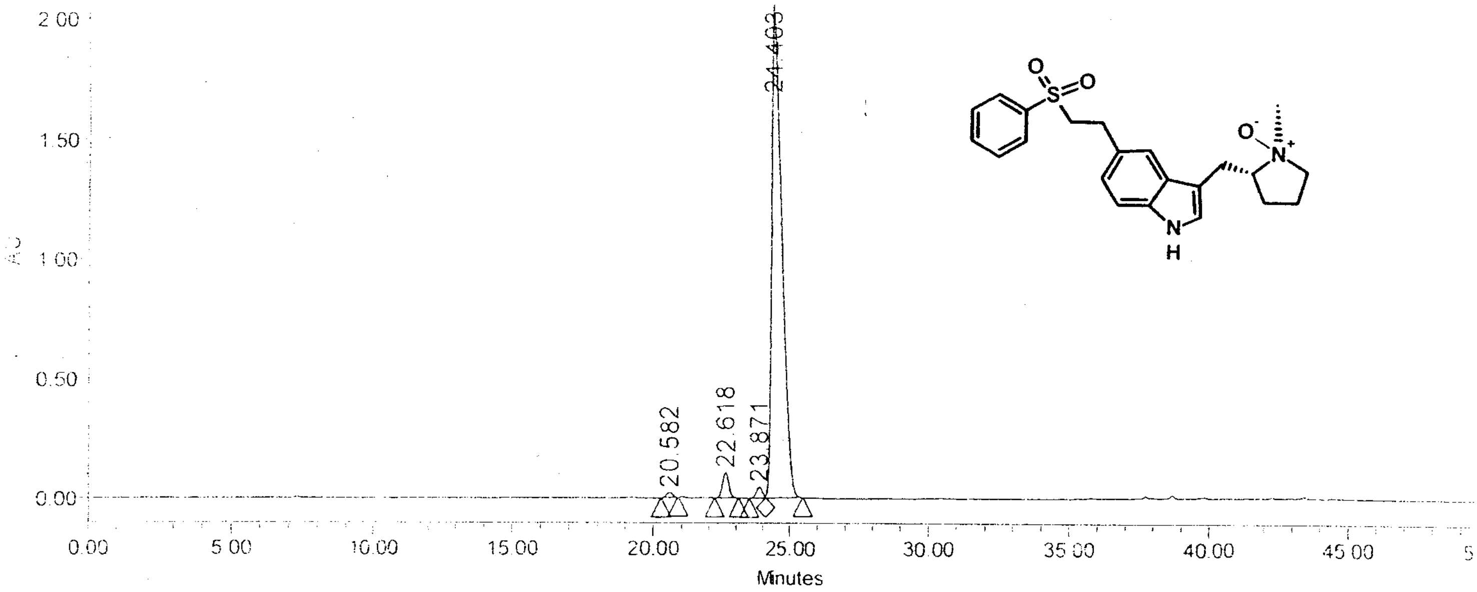

Peak Results

	RT (min)	Area (µV*sec)	% Area	RT Ratio	Name
1	20.58	329734	0.58	0.84	Peak6
2	22.62	1653197	2.93	0.93	Peak7
3	23.87	741458	1.32	0.98	Peak8
4	24.40	53651457	95.17	1.00	Eletriptan-N-Oxide-1

SAMPLE: chp-1708-19

Eletriptan N-Oxide-II
chp-1708-19

Solvent: cdcl3
Temp. 25.0 C / 298.1 K
Operator: apl
File: PROTON_01
VNMRS-500 "APLV500"

PULSE SEQUENCE
Relax. delay 1.000 sec
Pulse 45.0 degrees
Acq. time 1.809 sec
Width 9058.0 Hz
64 repetitions

OBSERVE H1, 499.8182339

DATA PROCESSING
FT size 32768
Total time 3 minutes

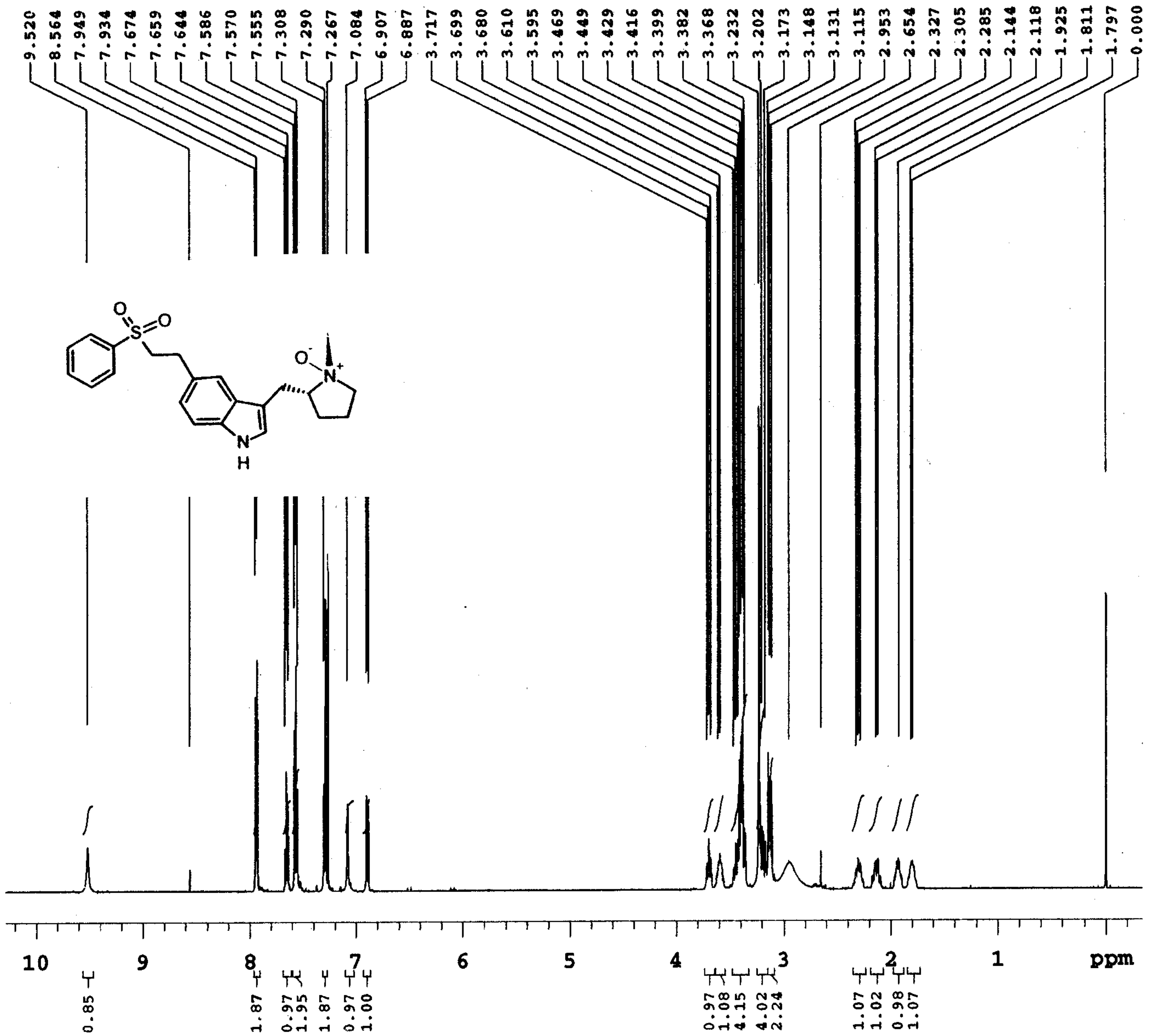

[illegible]/vnmrsys/data/2011/Jan | Sample: chp-1708-19_20110114 | File: PROTON_01

SAMPLE: chp-1761-25

Eletriptan N-oxide-II
chp-1761-25

Solvent: cdcl3
Temp. 25.0 C / 298.1 K
Operator: apl
File: DEPT_01
VNMRS-500 "APLV500"

PULSE SEQUENCE: DEPT
Relax. delay 1.000 sec
Pulse 90.0 degrees
Acq. time 1.022 sec
Width 31250.0 Hz
3264 repetitions

OBSERVE C13, 125.6793948
DECOUPLE H1, 499.8207327
Power 39 dB
on during acquisition
off during delay
WALTZ-16 modulated

DATA PROCESSING
Line broadening 0.5 Hz
FT size 65536
Total time 110 minutes

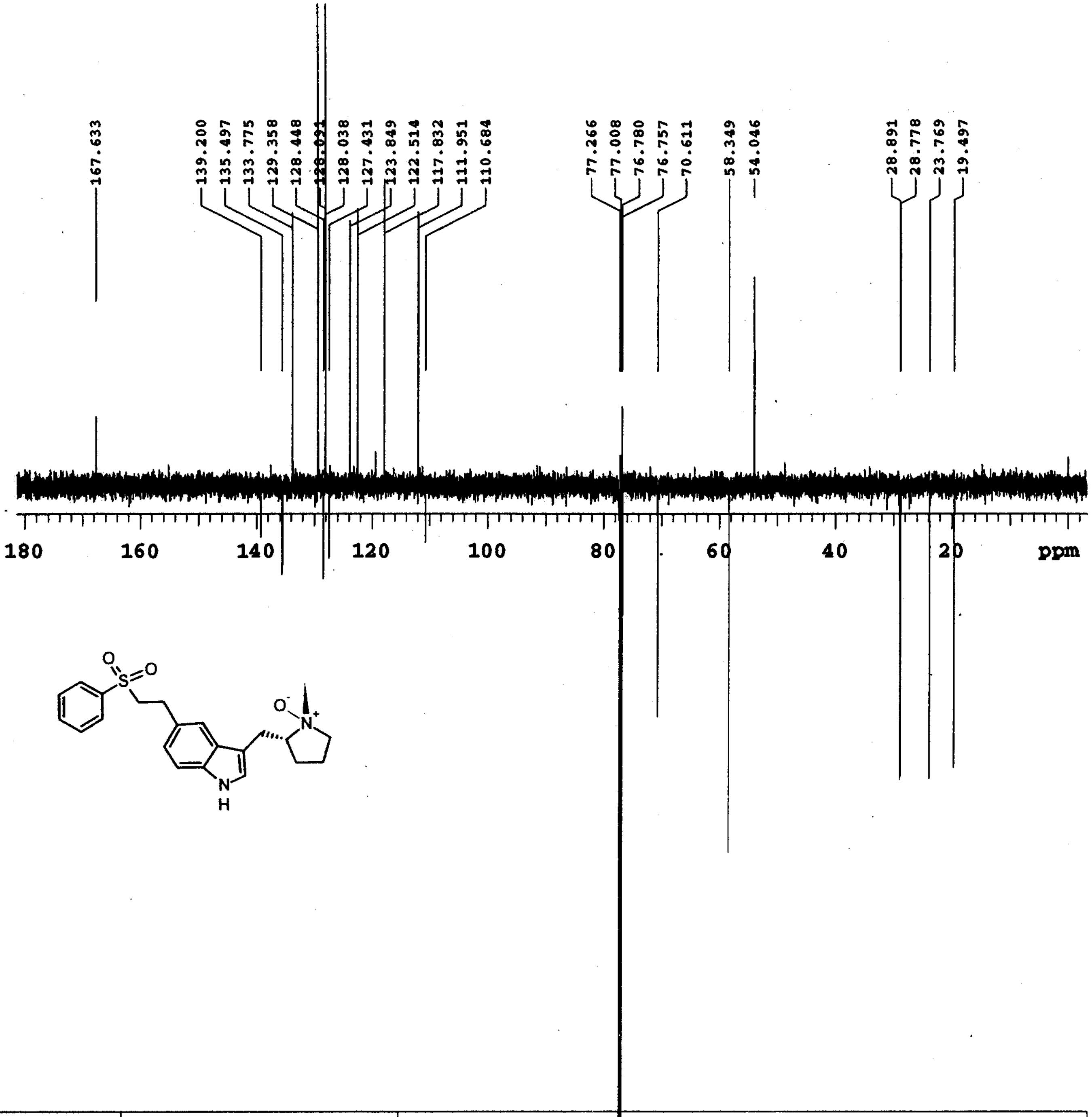

Archive directory: /home/aplchemsys/data/2011/May	Sample: chp-1761-25_20110521	File: DEPT_01

Analyst Version: 1.4.2
Acq. Time: 10:26
Acq. Date: Tuesday, December 14, 2010

APL RESEARCH CENTRE
ANALYTICAL RESEARCH DEPARTMENT

Sample Comment: B.NO:CHP(1708)19
Sample Name: ELETRIPTAN-N-OXIDE
Acq. File: DEC_14_CHP(1708)19_P_F_M.wiff

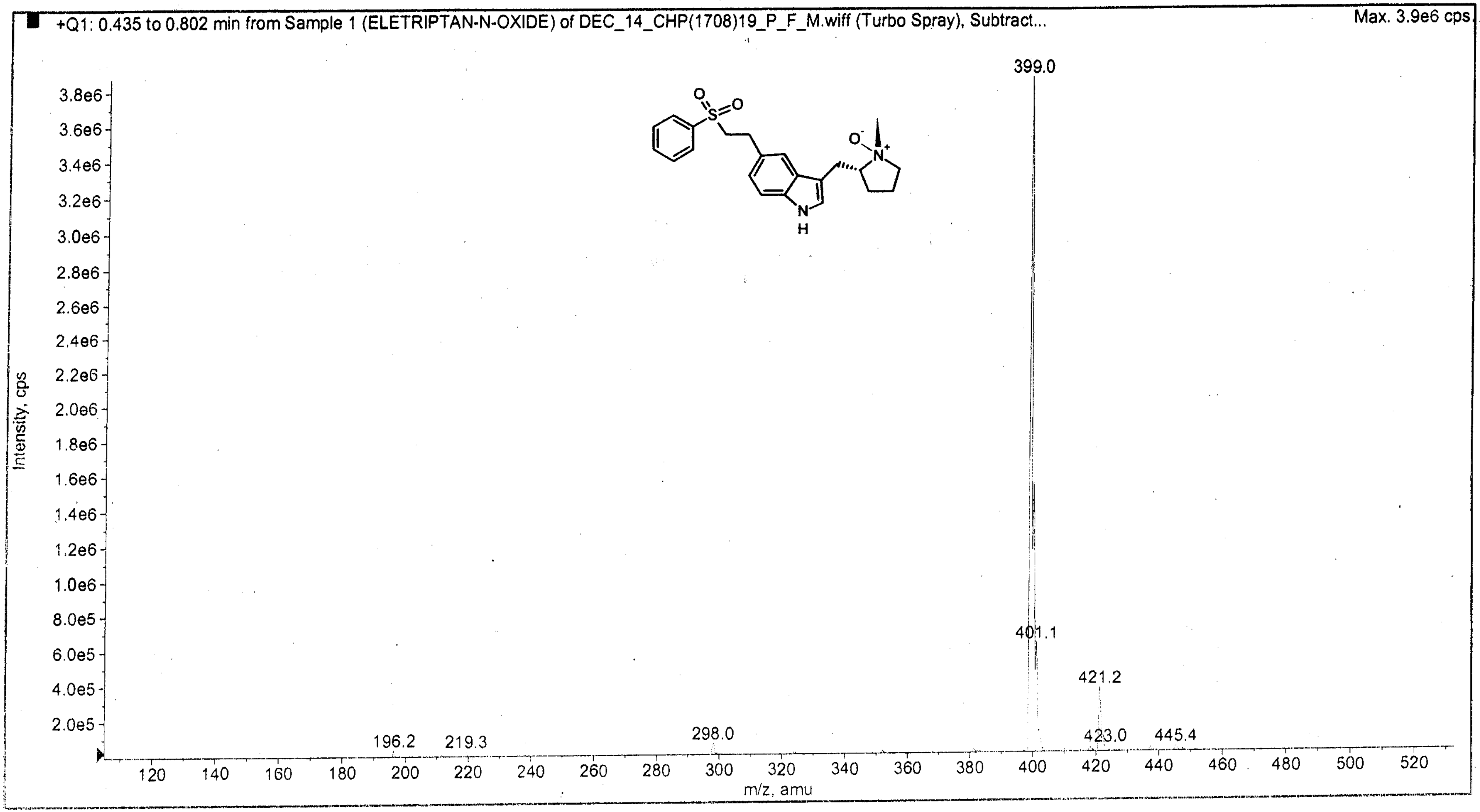

13

Period/Expt.: Period 1, Experiment 1
Scan Mode: Profile
Polarity/Scan Type: Positive Q1 MS

Analyst

Date: Monday, May 23, 2011

APL RESEARCH CENTRE

ARE_018

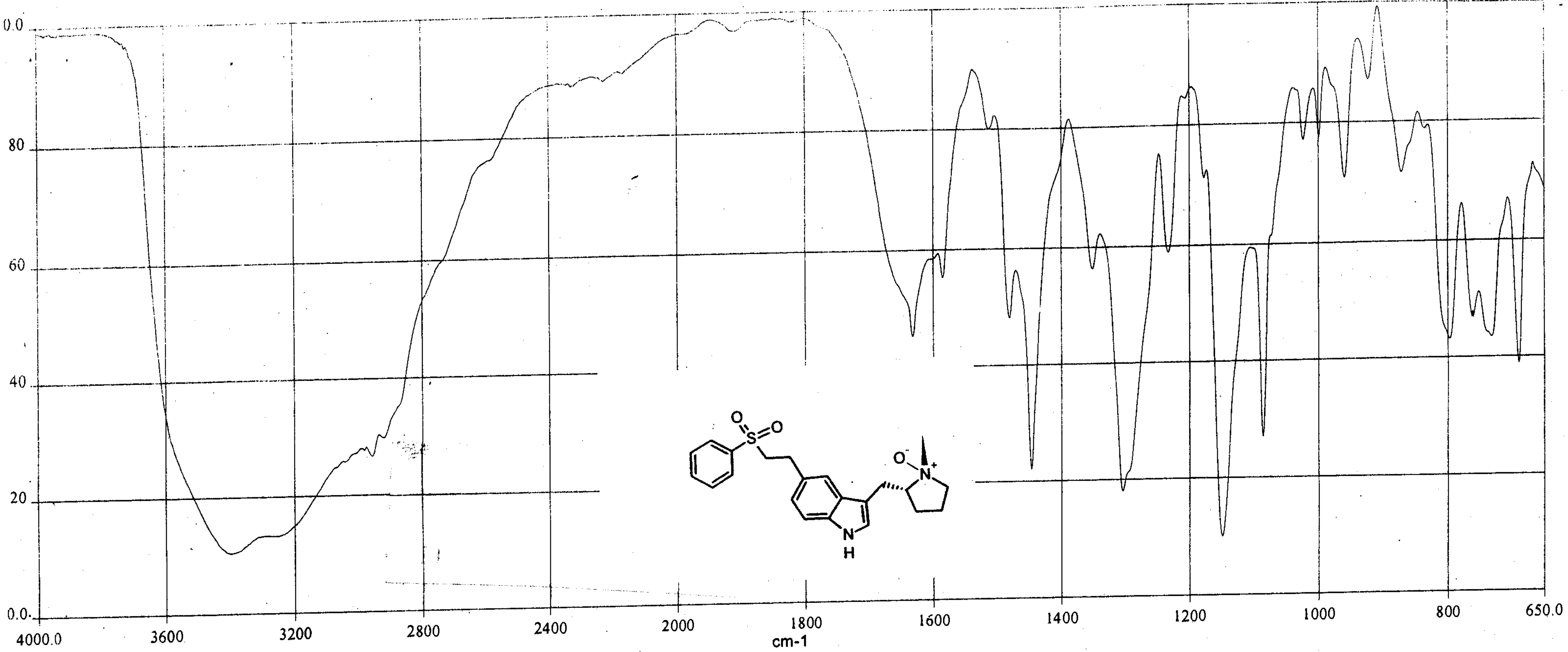

ELETRIPTAN N-OXIDE-2-CHP(1708)19.pk

ELETRIPTAN N-OXIDE-2-CHP(1708)19.002 3351 4000.00 650.00 10.00 99.23 4.00 %T 8 1.00
B.NO.: CHP(1708)19
REF 4000 99.12 2000 96.68 600
3400.65 10.42 2957.91 26.76 1910.97 97.05 1631.91 45.10 1584.52 55.18
1512.64 80.05 1480.71 48.01 1446.58 22.05 1351.07 56.32 1303.73 17.87
1232.22 58.94 1176.97 71.23 1148.74 9.98 1085.73 27.12 1022.98 77.17
998.80 77.55 959.58 70.76 922.45 87.02 871.23 71.49 796.65 43.13
760.98 46.69 731.55 43.44 689.35 38.85

Status

Filename: ELETRIPTAN N-OXIDE-2-CHP(1708)19.002
Date Created: Monday, May 23, 2011 11:15 AM India Standard Time
Analyst: Administrator
Description: B.NO.: CHP(1708)19
Comments:
SAMPLE: ELETRIPTAN N-OXIDE-2
PROJECT: ELETRIPTAN HBr
1.73 mg OF SAMPLE IN 388 mg OF KBr

Abscissa: (cm-1)
Start: 4000.00
End: 450.00
Interval: -1.000000

Ordinate: (%T)
Maximum: 100.00
Minimum: 10.00
Points: 3551

Instrument Model: Spectrum One

14

23/05/2011

APL RESEARCH CENTRE
ANALYTICAL RESEARCH DEPARTMEN'

INSTRUMENT ID : RCII_AE088

PROJECT NAME : MAR_2012\RCII_AE088

Sample ID	CHP(1708)19/Eletriptan -N-Oxide-2	Proc. Chnl. Descr :	W2996 PDA 225.0 nm [illegible]
Run Time	60.0 Minutes	Date Acquired :	3/29/2012 9:08:05 PM IST
Vial	32	Acq. Method Set :	Eletriptan_RS_MET
Injection	1	Date Processed :	3/30/2012 5:17:07 PM IST
Injection Volume	20.00 ul	Processing Method :	Eletriptan_RS_Pro

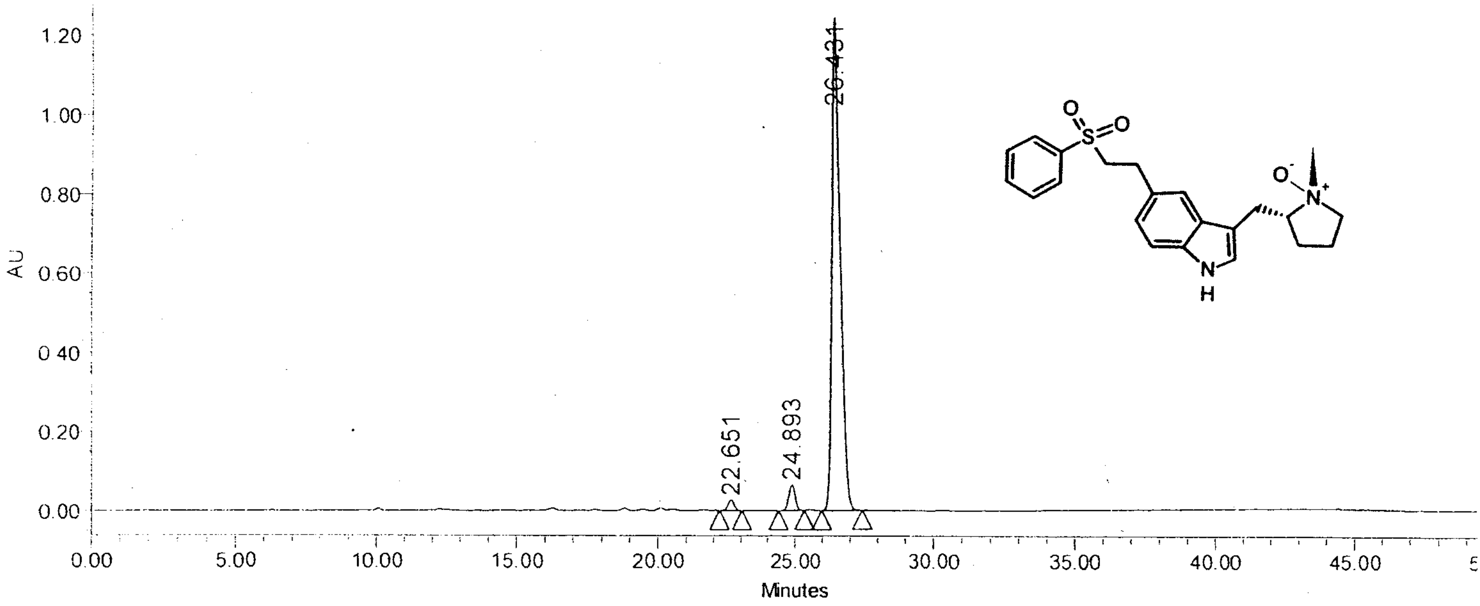

Peak Results

	RT (min)	Area (µV*sec)	% Area	RT Ratio	Name
1	22.65	417966	1.52	0.86	Peak7
2	24.89	1066365	3.87	0.94	Peak8
3	26.43	26087242	94.62	1.00	Eletriptan-N-Oxide-2

ELETRIPTAN UNK IMP RRT-0.87 CHP(1580)137 a.r.no: s-010041

(ETHYL INDOLE PYRROLIDINE IMPURITY) CDCl3

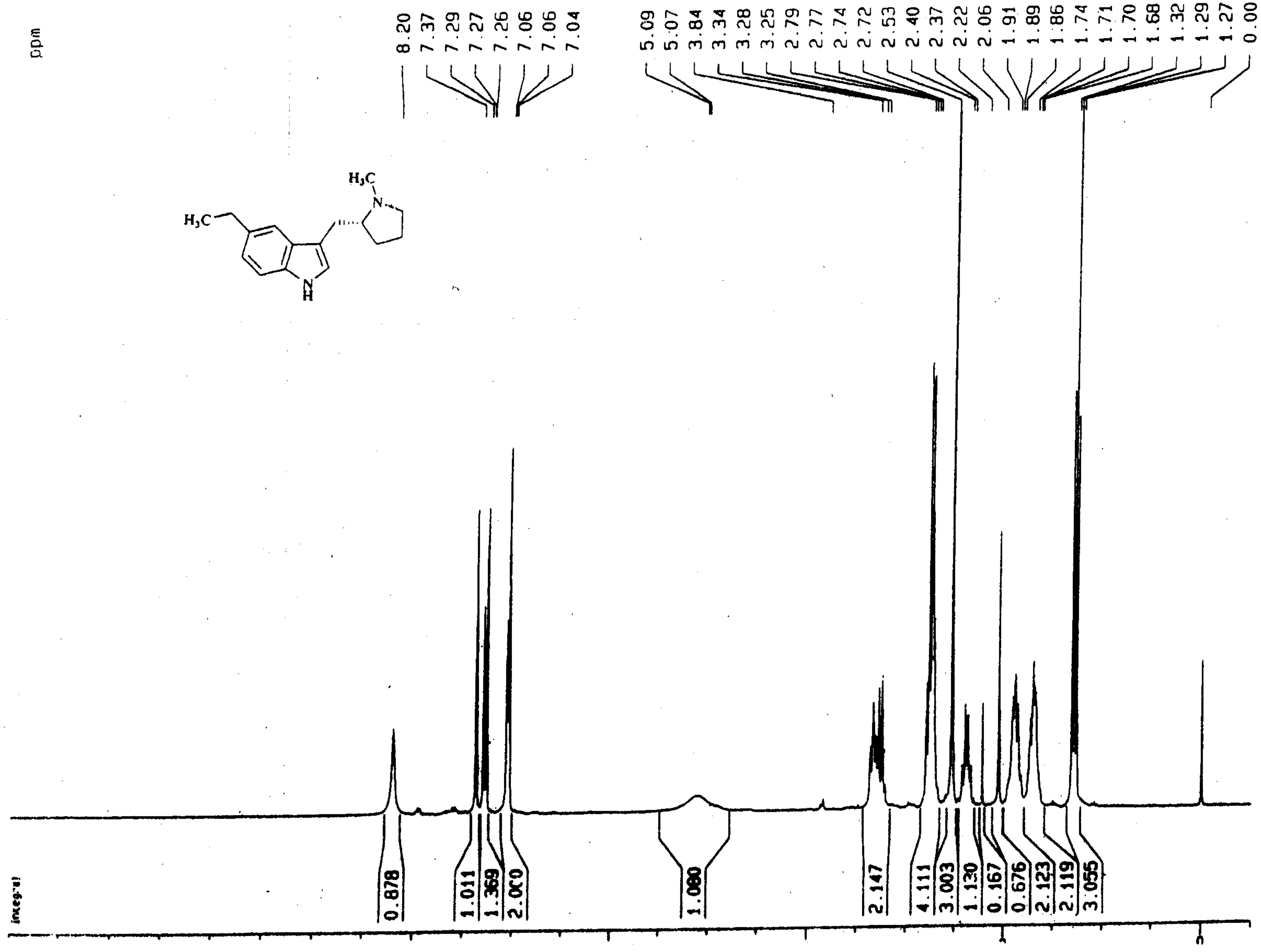

APL-RESEARCH CENTRE

Current Data Parameters

NAME	chp-1580-137
EXPNO	1
PROCNO	1

F2 - Acquisition Parameters

Date_	20100514
Time	10.44
INSTRUM	dpx300
PROBHD	5 mm BBO BB-
PULPROG	zg30
TD	32768
RG	362
SOLVENT	CDCl3
NS	16
AQ	1.8219508 sec
DW	55.600 usec
DE	6.00 usec
D1	2.00000000 sec
P1	6.75 usec
PL1	-6.00 dB
SFO1	300.1315006 MHz

============ CHANNEL f1 ===========

NUC1	1H

F2 - Processing parameters

SI	32768
SF	300.1300070 MHz
WDW	EM
LB	0.30 Hz
GB	0

1D NMR plot parameters

CX	20.50 cm
F1P	12.000 ppm
F2P	-0.500 ppm

16

SAMPLE: msk-1679-140

Ethyl Indole pyrrolidine imp
msk-1679-140

Solvent: cdcl3
Temp. 25.0 C / 298.1 K
Operator: apl
File: DEPT_01
VNMRS-500 "APLV500"

PULSE SEQUENCE: DEPT
Relax. delay 1.000 sec
Pulse 90.0 degrees
Acq. time 1.022 sec
Width 31250.0 Hz
9000 repetitions

OBSERVE C13, 125.6793948
DECOUPLE H1, 499.8207327
Power 39 dB
on during acquisition
off during delay
WALTZ-16 modulated

DATA PROCESSING
Line broadening 0.5 Hz
FT size 65536
Total time 5.1 hours

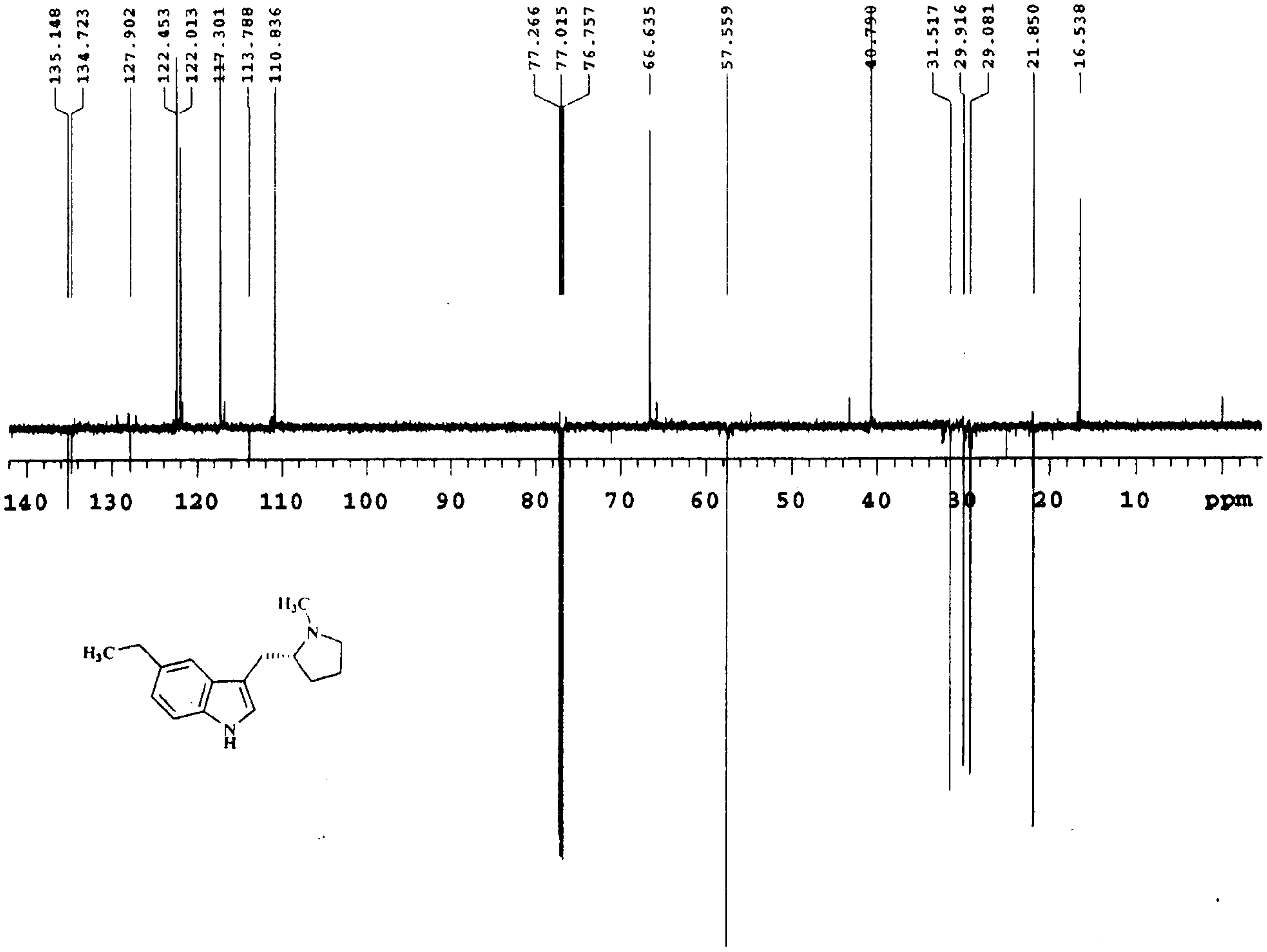

[illegible]/data/2011/Jun | Sample: msk-1679-140_20110623 | File: DEPT_01

17

Analyst Version: 1.4.2
Acq. Time: 09:24
Acq. Date: Friday, May 14, 2010

APL RESEARCH CENTRE
ANALYTICAL RESEARCH DEPARTMENT

ple Comment: BNO.:CHP(1580)137
Sample Name: ELETRIPTAN UNK IMP RRT AT
Acq. File: MAY_14_CHP(1580)137_P_F_M.wiff

(ETHYL INDOLE PYRROLIDINE IMPURITY)

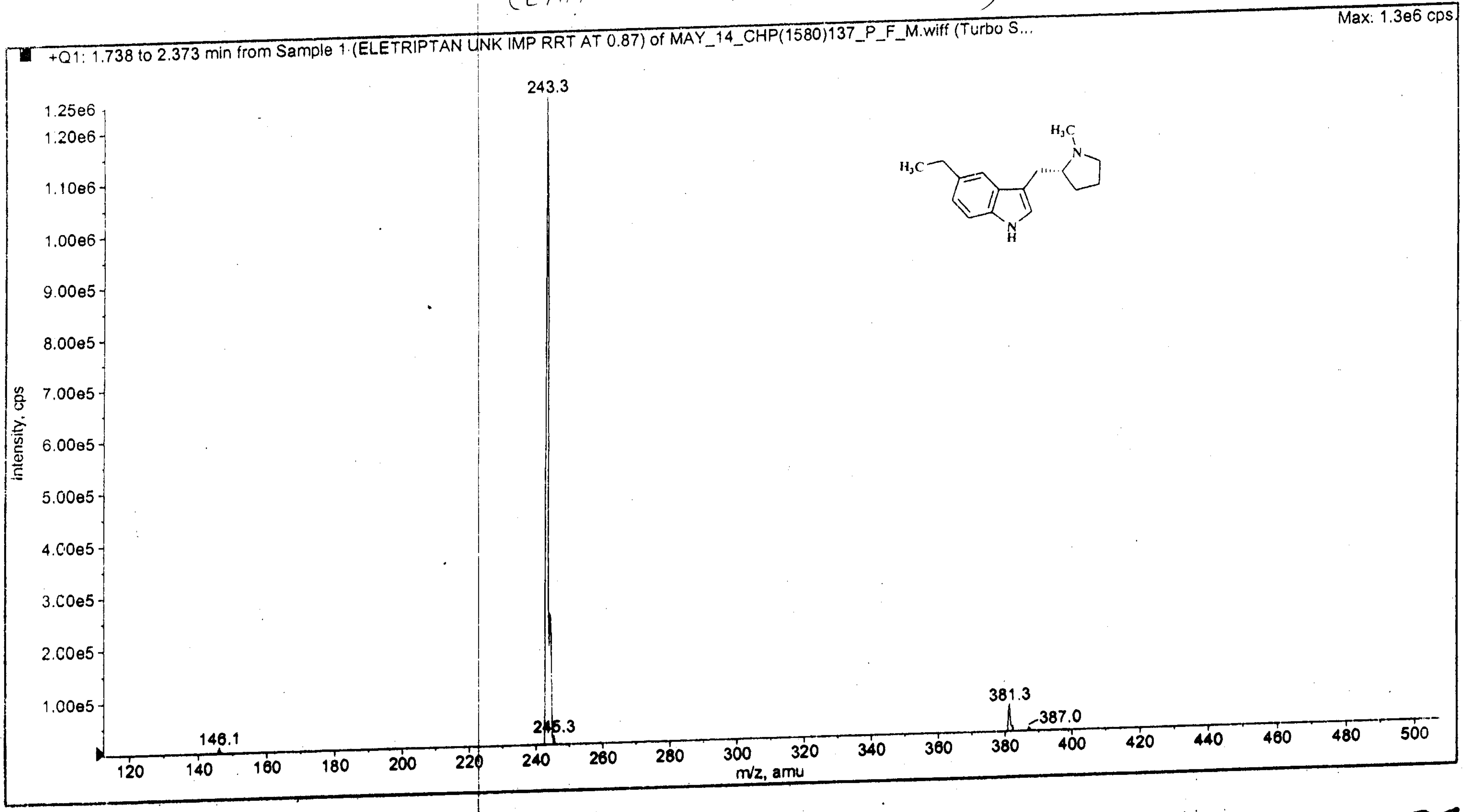

18

Analyst

Period/Expt.: Period 1, Experiment 1
Scan Mode: Profile
Polarity/Scan Type: Positive Q1 MS

Date: Monday, May 23, 2011

APL RESEARCH CENTRE

ARE_018

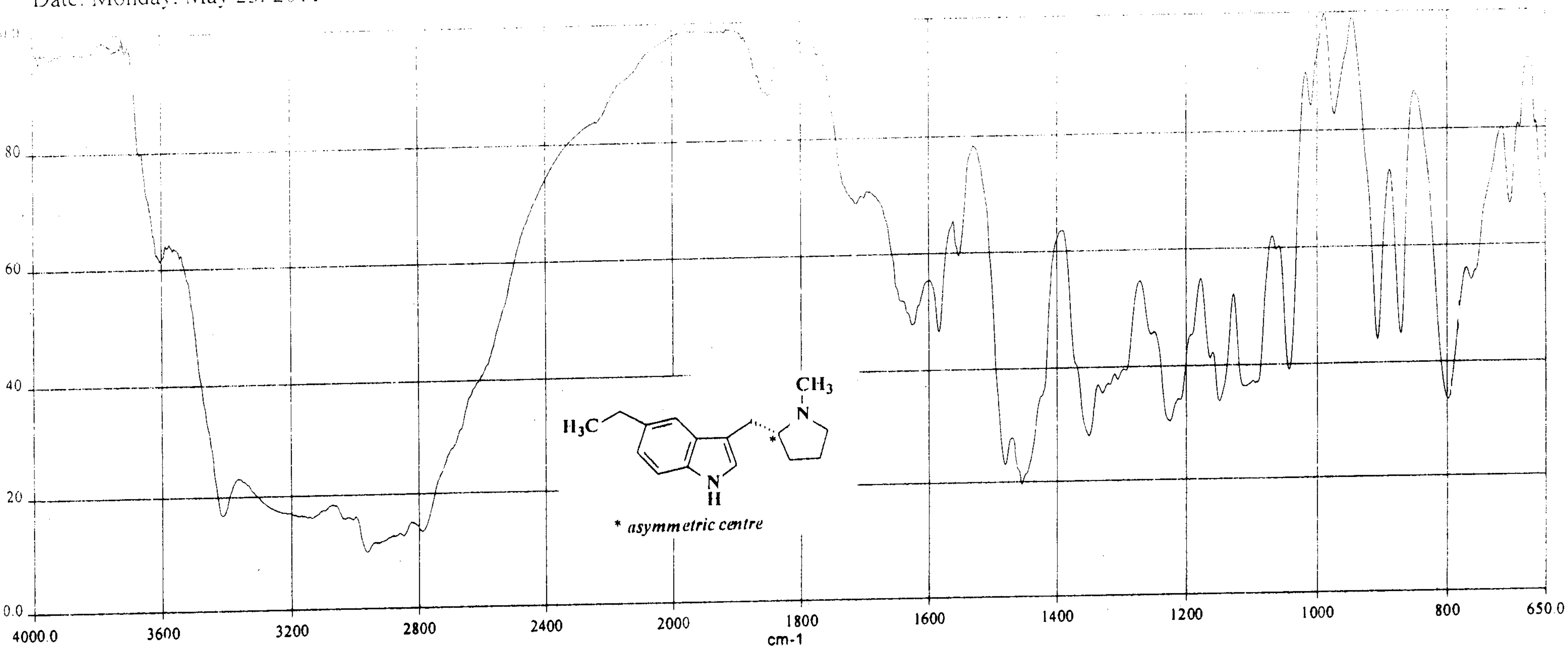

(ETHYL INDOLE PYRROLIDINE IMPURITY)

Status

Filename: ETHYL INDOLE PROLINE-MSK(1679)140.002
Date Created: Monday, May 23, 2011 10:07 AM India Standard Time
Analyst: Administrator
Description: B.NO.: MSK(1679)140
Comments:
SAMPLE: ETHYL INDOLE PROLINE
PROJECT: ELETRIPTAN HBr
NEAT BG

Abscissa: (cm-1)
Start: 4000.00
End: 490.00
Interval: -1.000000

Ordinate: (%T)
Maximum: 100.00
Minimum: 10.00
Points: 3511

Instrument Model: Spectrum One
Instrument Serial Number: 53009.

ETHYL INDOLE PROLINE-MSK(1679)140.pk

ETHYL INDOLE PROLINE-MSK(1679)140.002 3351 4000.00 650.00 10.00 99.70 4.00 %T 8 1.00
B.NO.: MSK(1679)140
REF 4000 99.31 2000 97.99 600
3981.16 95.31 3948.26 95.95 3751.20 97.03 3710.32 96.78 3604.33 61.33
3411.30 16.76 3134.92 16.05 2963.40 9.99 2790.47 13.41 1850.00 87.61
1712.77 68.76 1625.98 47.88 1583.58 46.59 1552.04 59.42 1480.82 22.94
1454.98 19.52 1350.20 28.06 1329.68 35.64 1224.45 30.45 1148.67 33.84
1110.97 36.37 1042.31 38.97 1009.36 84.14 973.21 82.70 906.43 43.94
870.78 44.90 798.24 33.59 762.14 54.00 703.99 66.91

19

23/05/2011

APL RESEARCH CENTRE
ANALYTICAL RESEARCH DEPARTMENT

INSTRUMENT ID : RCII_AE088 PROJECT NAME : MAR_2012\RCII_AE088

Sample ID	ELE(1130)42//Desbromo indole pyrrolidine imp	Proc. Chnl. Descr :	W2996 PDA 225.0 nm
Run Time	60.0 Minutes	Date Acquired :	3/29/2012 6:04:57 PM IST
Vial	29	Acq. Method Set :	Eletriptan_RS_MET
Injection	1	Date Processed :	3/30/2012 9:33:03 AM IST
Injection Volume :	20.00 ul	Processing Method :	Eletriptan_RS_Pro

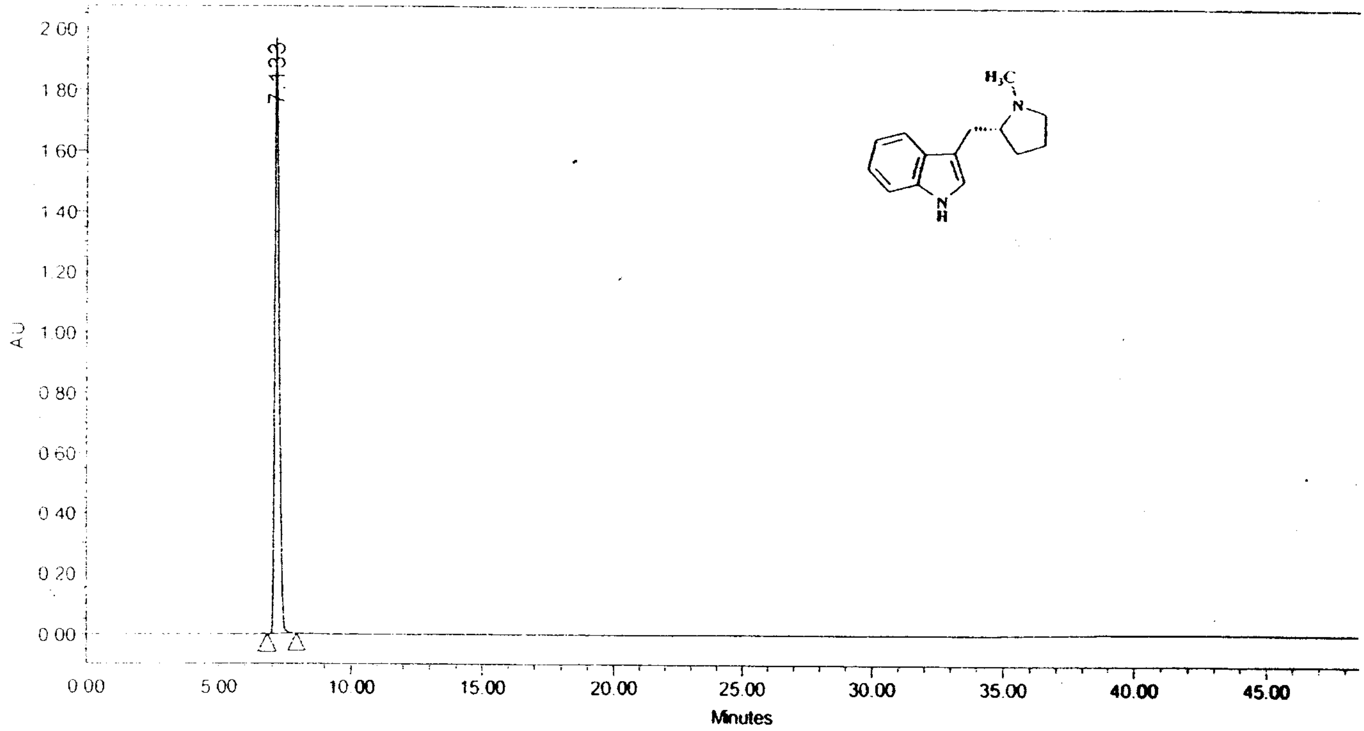

Peak Results

	RT (min)	Area (µV*sec)	% Area	Name
1	7.13	23663578	100.00	Desbromo indole pyrrolidine imp

DESBROMO BIP ELE (1130) 42 A.R.NO: S-010067

CDC13

(DES BROMO INDOLE PYRROLIDINE IMPURITY)

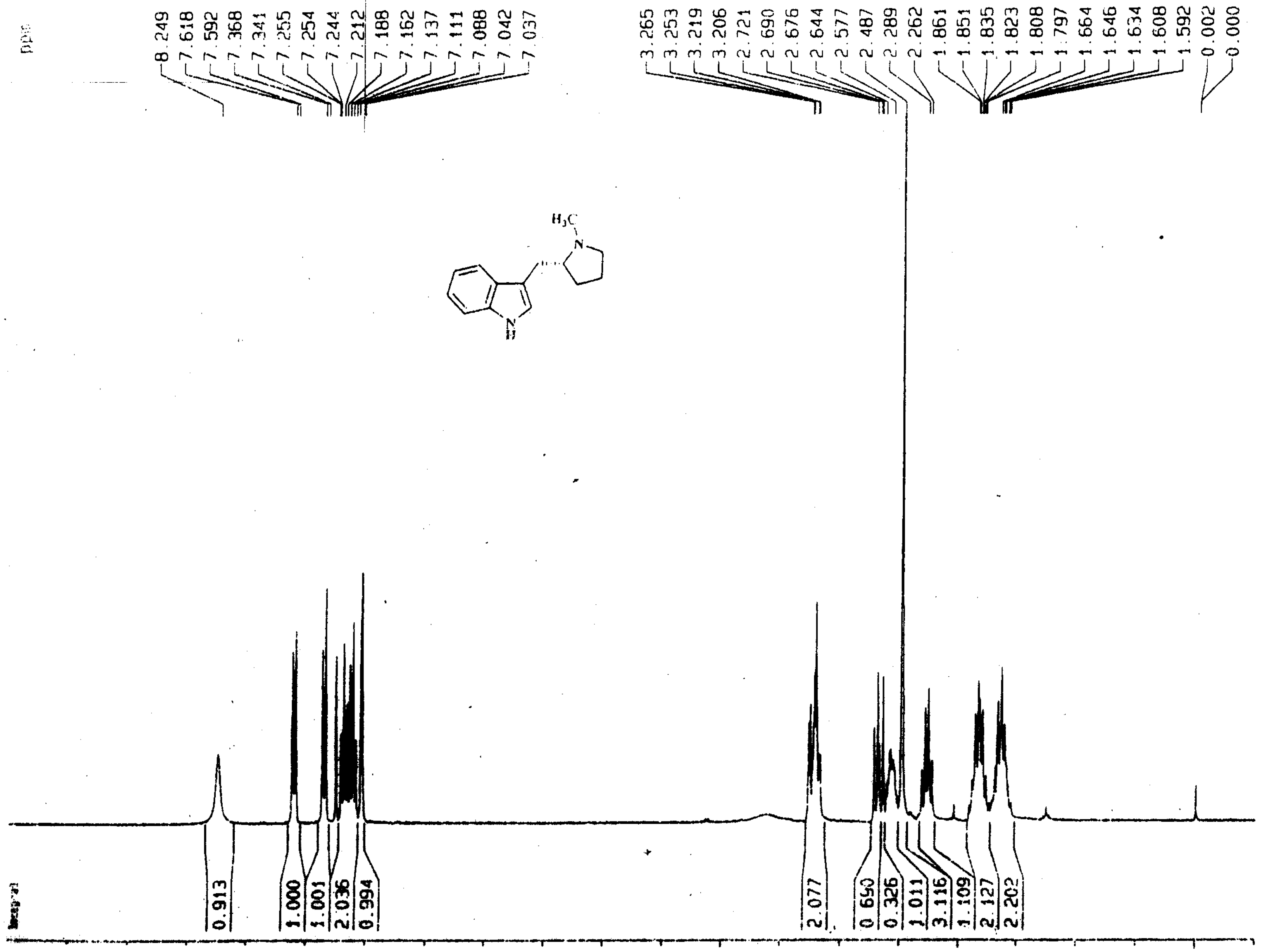

APL-RESEARCH CENTRE

Current Data Parameters

NAME ele-1130-42

PROCNO 1

F2 - Acquisition Parameters

Date_ 20100621

Time 16.49

INSTRUM dpx300

PROBHD 5 mm BBO BB-

PULPROG zg30

TD 32768

RG 362

SOLVENT CDC13

NS 16

AQ 1.8219508 sec

DW 55.600 usec

DE 6.00 usec

D1 2.00000000 sec

P1 6.75 usec

PL1 -6.00 dB

SFO1 300.1315006 MHz

============ CHANNEL f1 ==========

NUC1 1H

F2 - Processing parameters

SI 32768

SF 300.1300083 MHz

WDW EM

LB 0.30 Hz

GB 0

1D NMR plot parameters

CX 20.50 cm

F1P 10.000 ppm

F2P -0.500 ppm

21

(DES BROMO INDOLE PYRROLIDINE IMPURITY)

PENDANT-NMR of DESBROMO INDOLE PROLINE ELE (1130) 42

CDC13

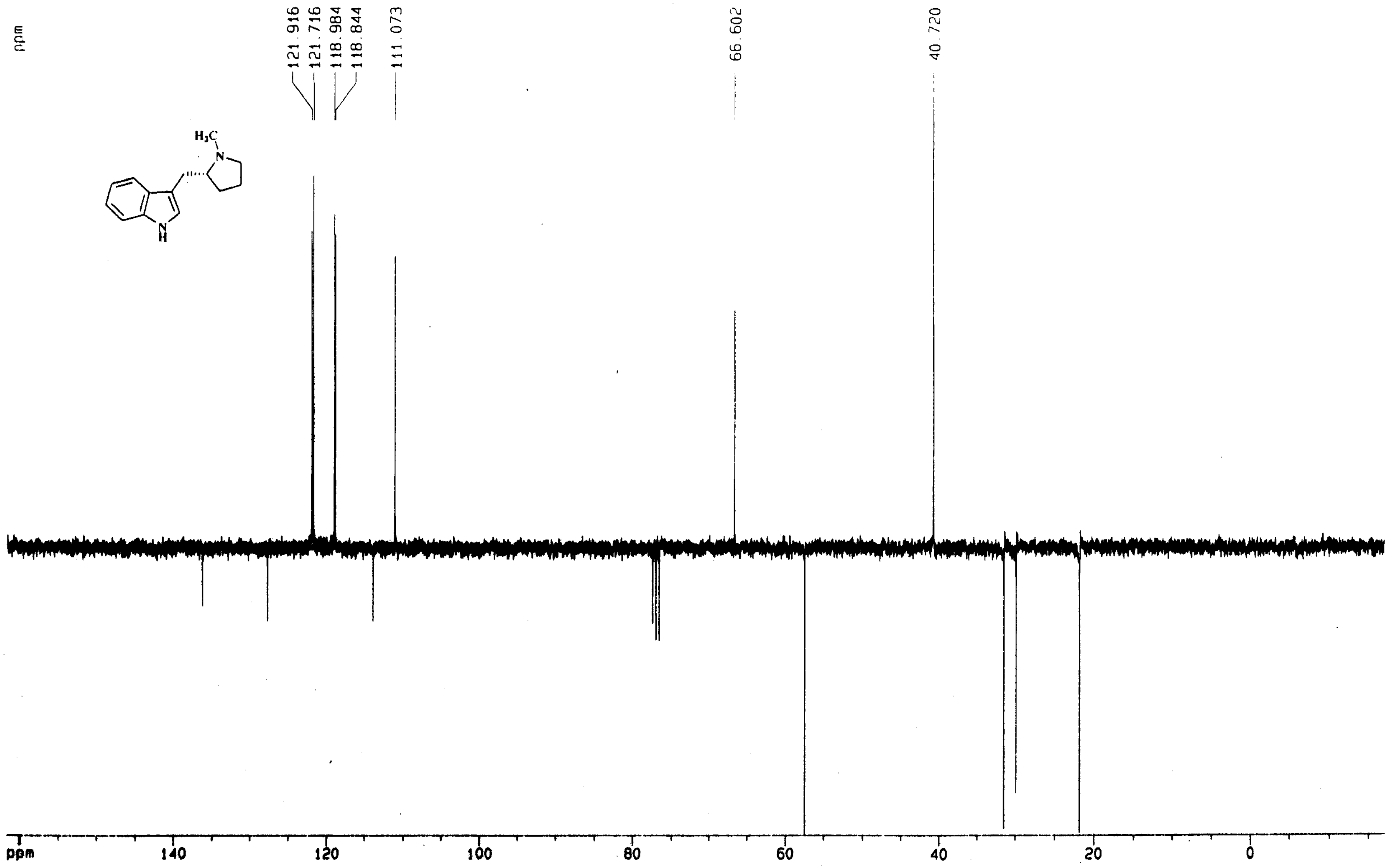

Analyst Version: 1.4.2
Acq. Time: 10:29
Acq. Date: Thursday, October 07, 2010

APL RESEARCH CENTRE
ANALYTICAL RESEARCH DEPARTMENT

[illegible]nple Comment: B.NO:ELE(1130)42
Sample Name: DESBROMO BIP
Acq. File: OCT_07_ELE(1130)42_P_F_M.wiff

(DES BROMO INDOLE PYRROLIDINE IMPURITY)

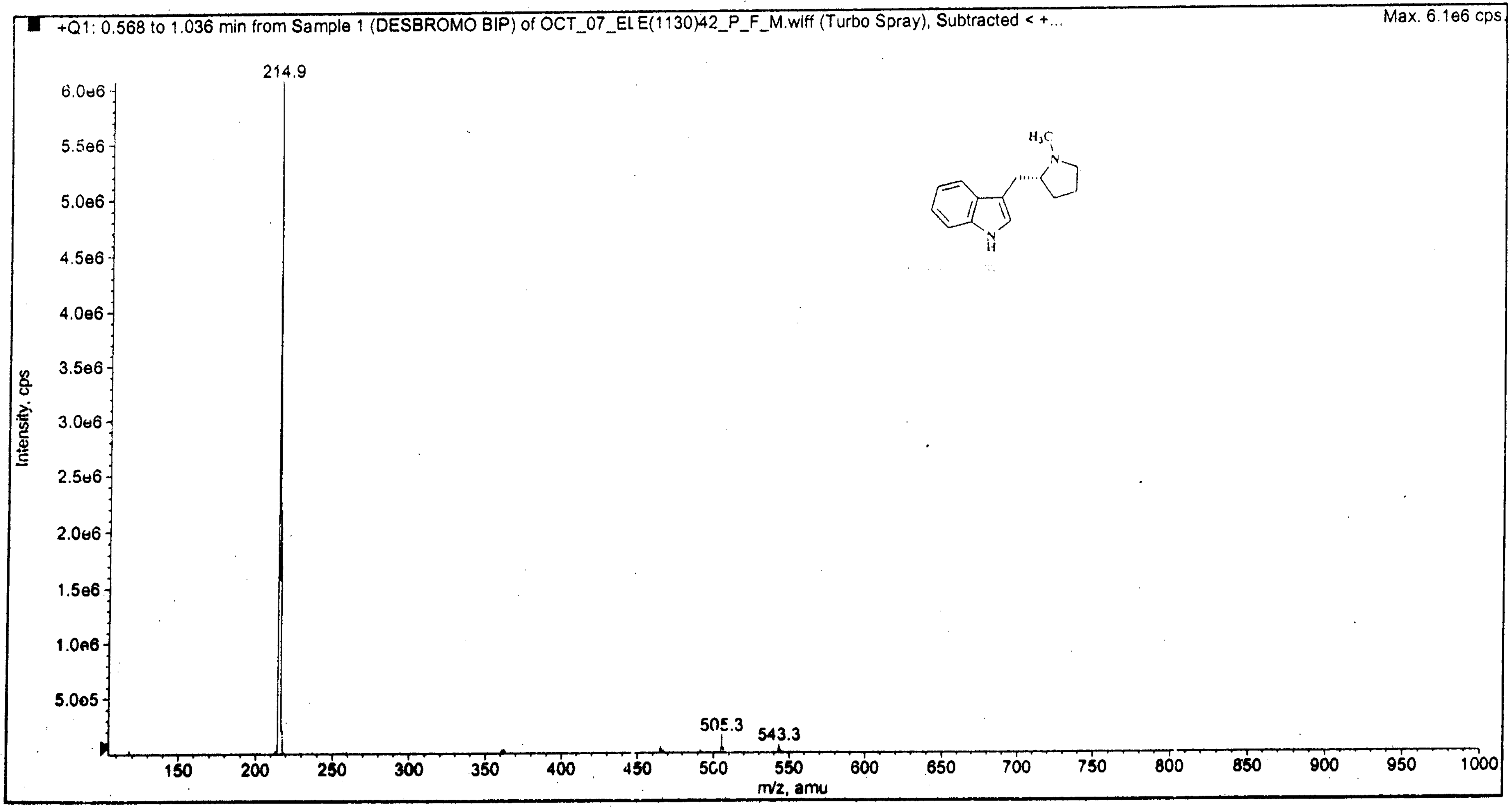

Period/Expt.: Period 1, Experiment 1
Scan Mode: Profile
Polarity/Scan Type: Positive Q1 MS

Analyst

Date: Monday, May 23, 2011

APL RESEARCH CENTRE

ARE_018

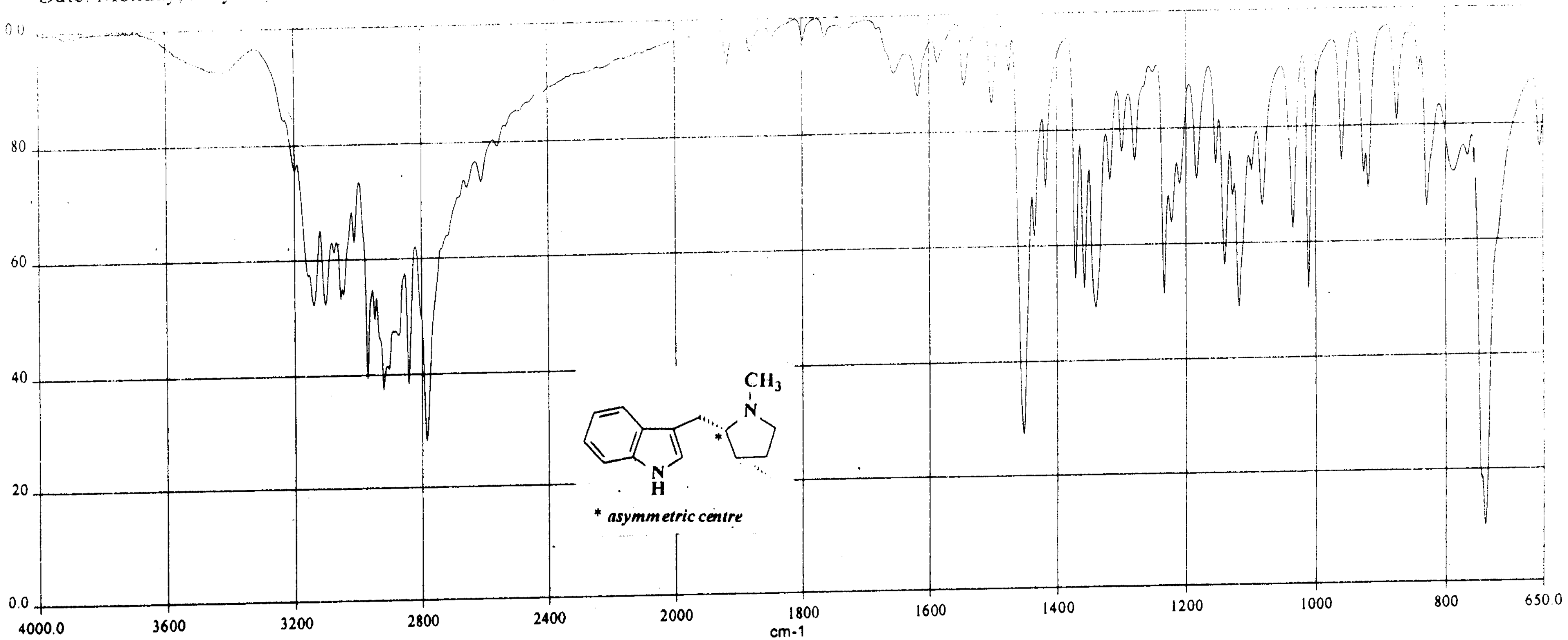

24

Status (DES BROMO INDOLE PYRROLIDINE IMPURITY)

Filename: DESBROMO ANALOG OF ELETRIPTAN-ELE(1130)42.002
Date Created: Monday, May 23, 2011 11:27 AM India Standard Time
Analyst: Administrator
Description: B.NO.: ELE(1130)42
Comments:
SAMPLE: DESBROMO ANALOG OF ELETRIPTAN
PROJECT: ELETRIPTAN HBr
1.86 mg OF SAMPLE IN 397 mg OF KBr

Abscissa: (cm-1)
Start: 4000.00
End: 450.00
Interval: -1.000000

Ordinate: (%T)
Maximum: 100.00
Minimum: 10.00
Points: 3551

Spectrum One

DESBROMO ANALOG OF ELETRIPTAN-ELE(1130)42.pk

DESBROMO ANALOG OF ELETRIPTAN-ELE(1130)42.002 3351 4000.00 650.00 10.00 99.90 4.00 %
B.NO.: ELE(1130)42
REF 4000 99.77 2000 96.53 600
3428.53 92.47 3199.11 75.57 3139.70 52.25 3102.35 52.28 3074.80 61.25
3054.38 53.20 3045.28 53.99 3012.40 63.04 2972.03 39.40 2949.87 49.62
2922.95 37.49 2842.99 38.49 2785.71 28.46 2658.41 72.39 2612.72 73.31
2562.21 79.26 1917.71 92.40 1880.99 94.60 1847.11 96.93 1798.60 96.14
1762.74 96.75 1655.70 90.30 1618.30 86.32 1586.64 92.07 1545.50 88.02
1502.27 84.90 1474.64 90.31 1451.99 27.56 1434.30 61.90 1417.20 70.32
1371.23 54.24 1357.86 52.68 1340.02 49.14 1317.86 71.40 1299.31 76.19
1278.73 74.62 1250.68 89.75 1233.05 51.25 1221.62 63.97 1209.47 70.52
1183.05 71.36 1153.03 73.83 1138.89 56.16 1126.62 68.37 1116.89 48.99
1097.67 72.62 1081.02 66.71 1034.69 62.53 1010.61 52.11 959.44 74.18
925.98 71.90 919.03 69.34 875.71 80.89 841.40 89.33 828.78 66.02
787.66 71.97 765.86 74.59 739.23 9.95 655.30 76.04

23/05/2011

APL RESEARCH CENTRE
ANALYTICAL RESEARCH DEPARTMEN'

INSTRUMENT ID RCII_AE088 PROJECT NAME MAR_2012\RCII_AE088

Sample ID	CHP(1580)137/ Ethyl indole pyrrolidine imp	Proc. Chnl. Descr	W2996 PDA 225.0 nm
		Date Acquired	3/29/2012 7:05:59 PM IS'
Run Time	60.0 Minutes	Acq Method Set	Eletriptan_RS_MET
Vial	30		
Injection	1	Date Processed	3/30/2012 5:15:44 PM IS
Injection Volume	20.00 ul	Processing Method	Eletriptan_RS_Pro

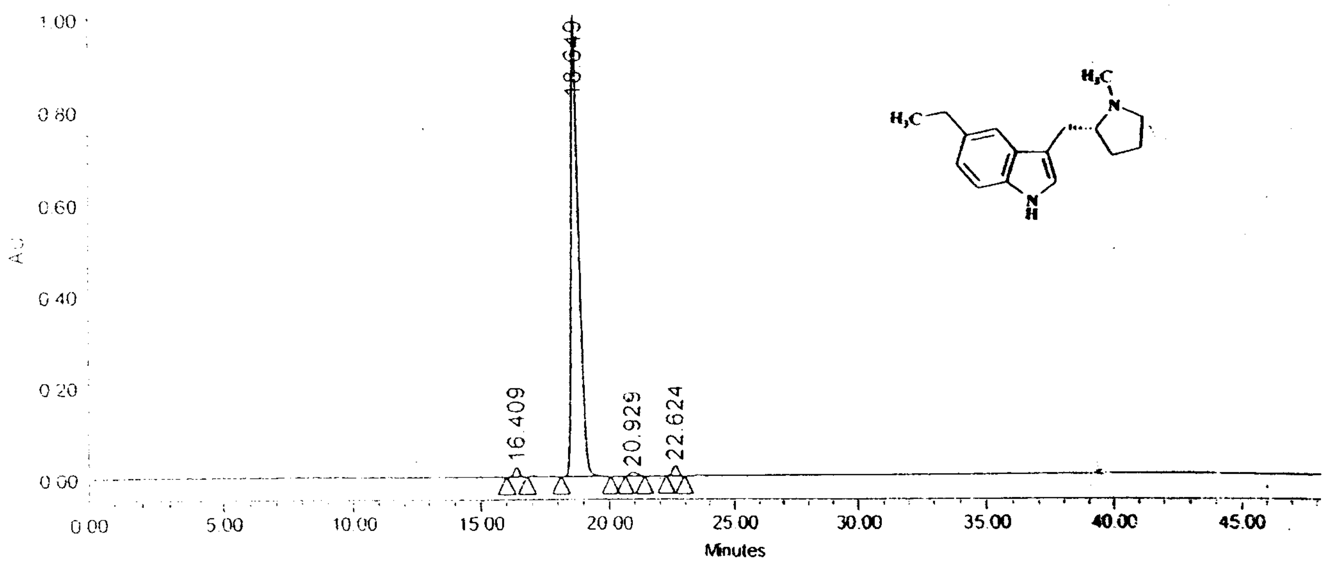

Peak Results

	RT (min)	Area (µV*sec)	% Area	RT Ratio	Name
1	16.41	301273	1.44	0.88	Peak5
2	18.65	20062648	95.88	1.00	Ethyl indole pyrrolidine imp
3	20.93	200745	0.96	1.12	Peak6
4	22.62	360622	1.72	1.21	Peak7

ELETRIPTAN STG-II UNK IMP RT-21.20 KSA (1637) 65 A.R.NO: S-010077

(ELETRIPTAN METHOXY IMPURITY) DMSO-d6

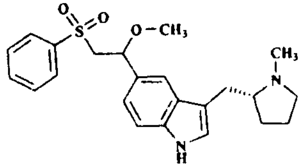

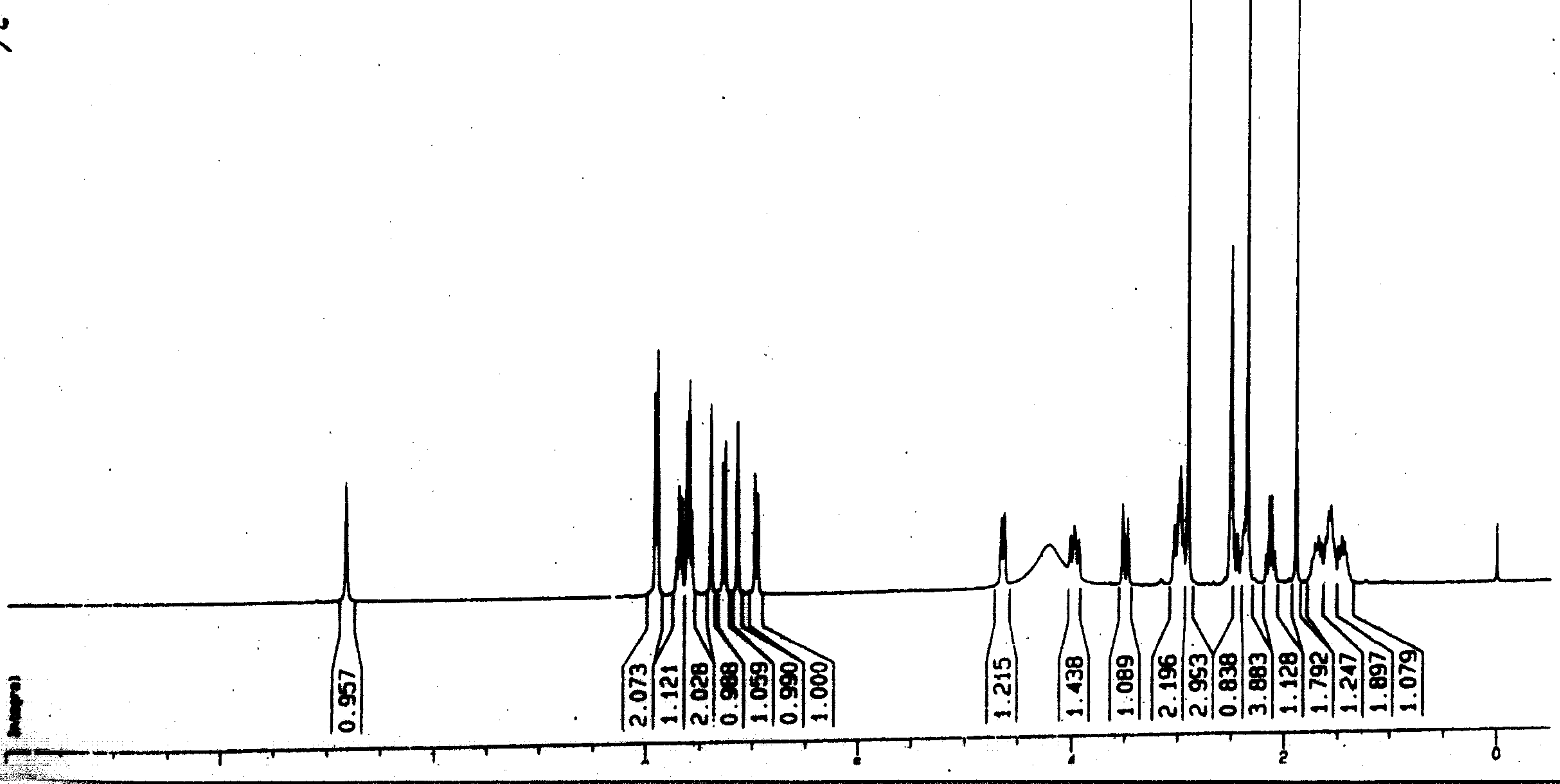

APL-RESEARCH CENTRE

Current Data Parameters

NAME	ksa-1637-ele
EXPNO	1
PROCNO	1

F2 - Acquisition Parameters

Date_	20100717
Time	10.33
INSTRUM	dpx300
PROBHD	5 mm BBO BB-
PULPROG	zg30
TD	32768
RG	287.4
SOLVENT	DMSO
NS	16
AQ	1.8219508 sec
DW	55.600 usec
DE	6.00 usec
D1	2.00000000 sec
P1	6.75 usec
PL1	-6.00 dB
SFO1	300.1315006 MHz

======== CHANNEL f1 ========

NUC1	1H

F2 - Processing parameters

SI	32768
SF	300.1300004 MHz
WDW	EM
LB	0.30 Hz
GB	0

1D NMR plot parameters

CX	20.50 cm
F1P	14.000 ppm
F2P	-0.500 ppm

92

PENDANT-NMR of METHOXY ELETRIPTAN KSA(1637)66 A.R.NO: S-010077

DMSO-d6

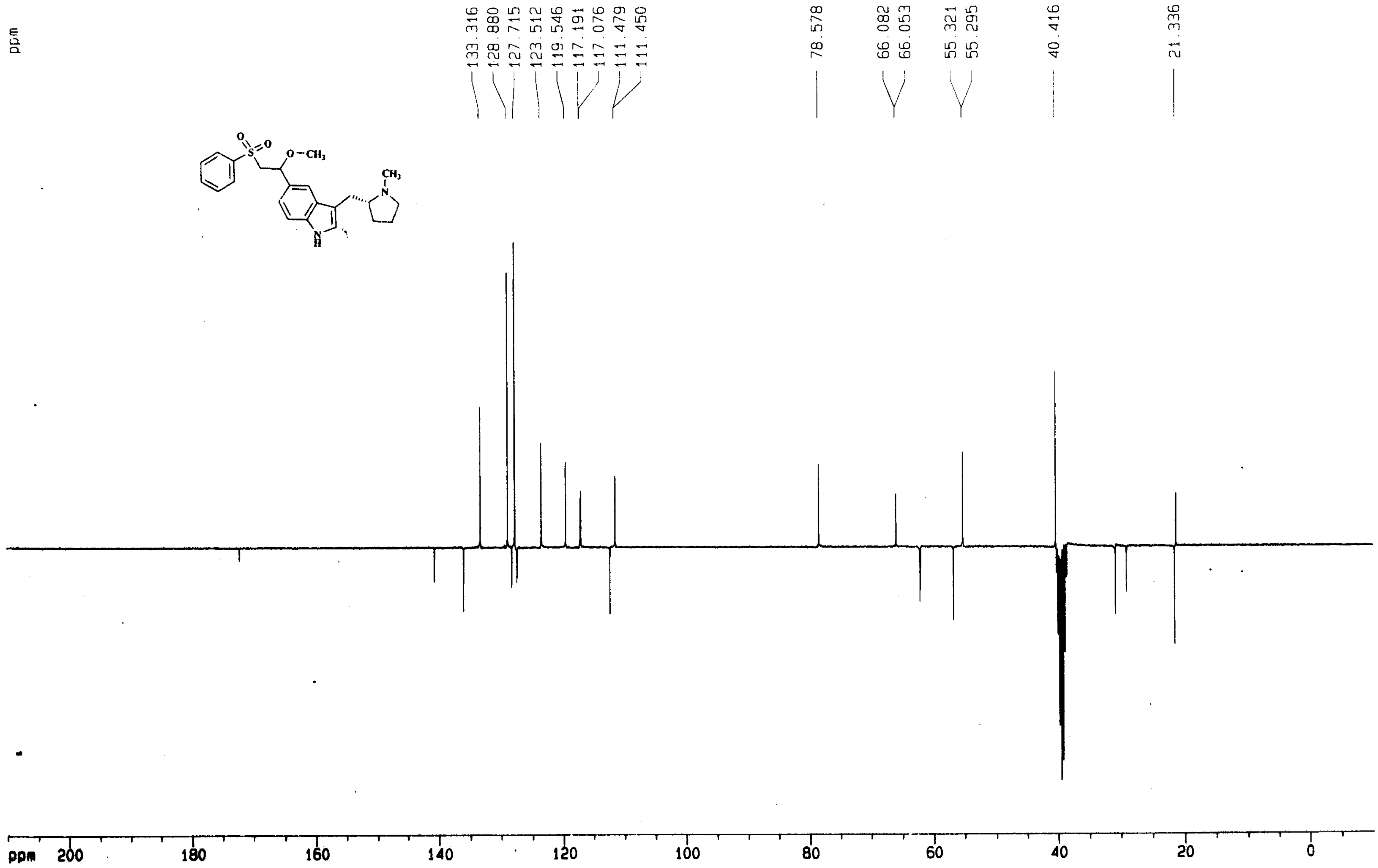

27

Analyst Version: 1.4.2
Acq. Time: 10:52
Acq. Date: Saturday, July 17, 2010

APL RESEARCH CENTRE
ANALYTICAL RESEARCH DEPARTMENT

Sample Comment: BNO.:KSA(1637)__
Sample Name: ELETRIPTAN IMP RT-21.20min
Acq. File: JUL_17_KSA(1637)__P_F_M.wiff

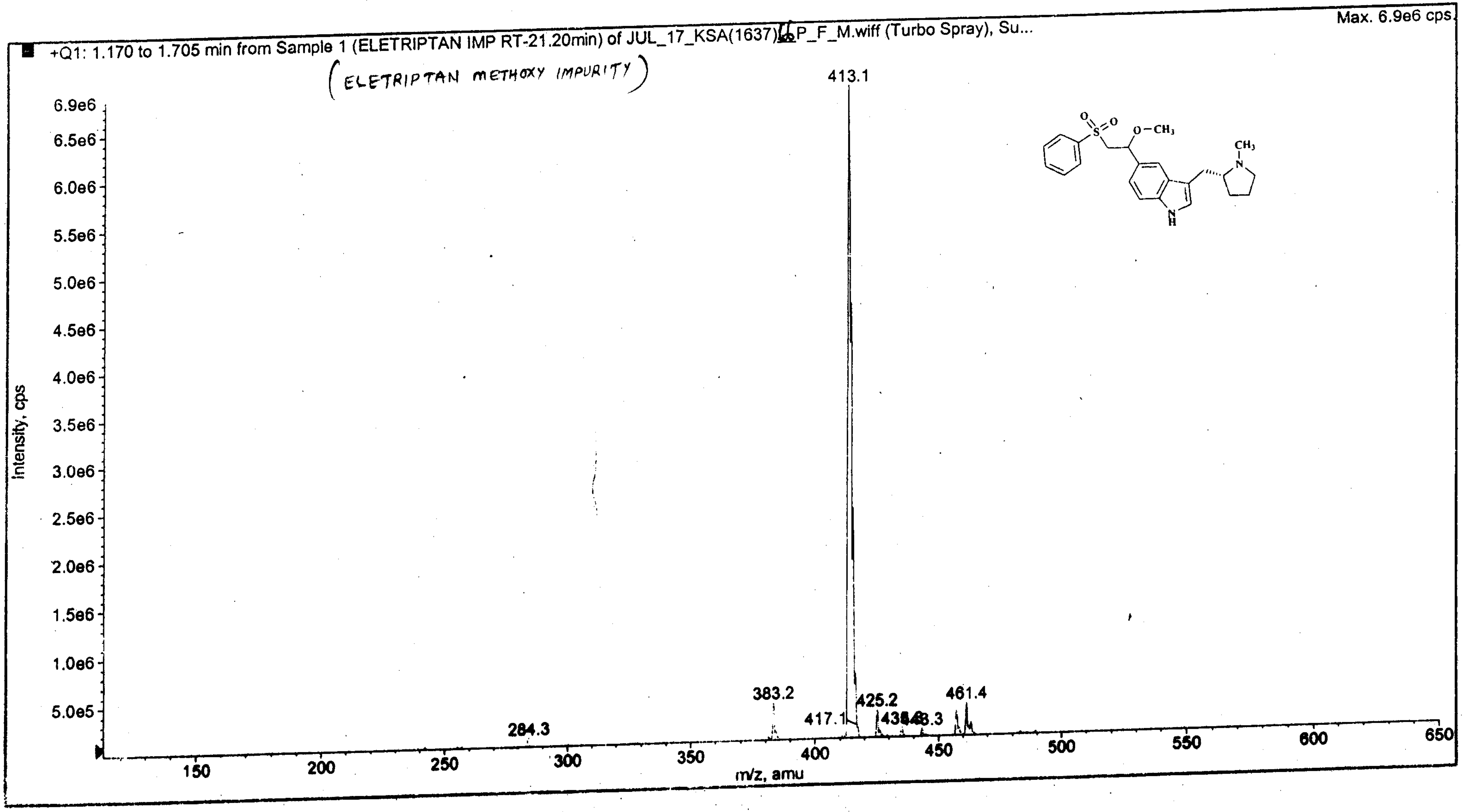

28

Analyst

Period/Expt.: Period 1, Experiment 1
Scan Mode: Profile
Polarity/Scan Type: Positive Q1 MS

Date: Saturday, March 17, 2012 APL RESEARCH CENTRE RCII_AE009

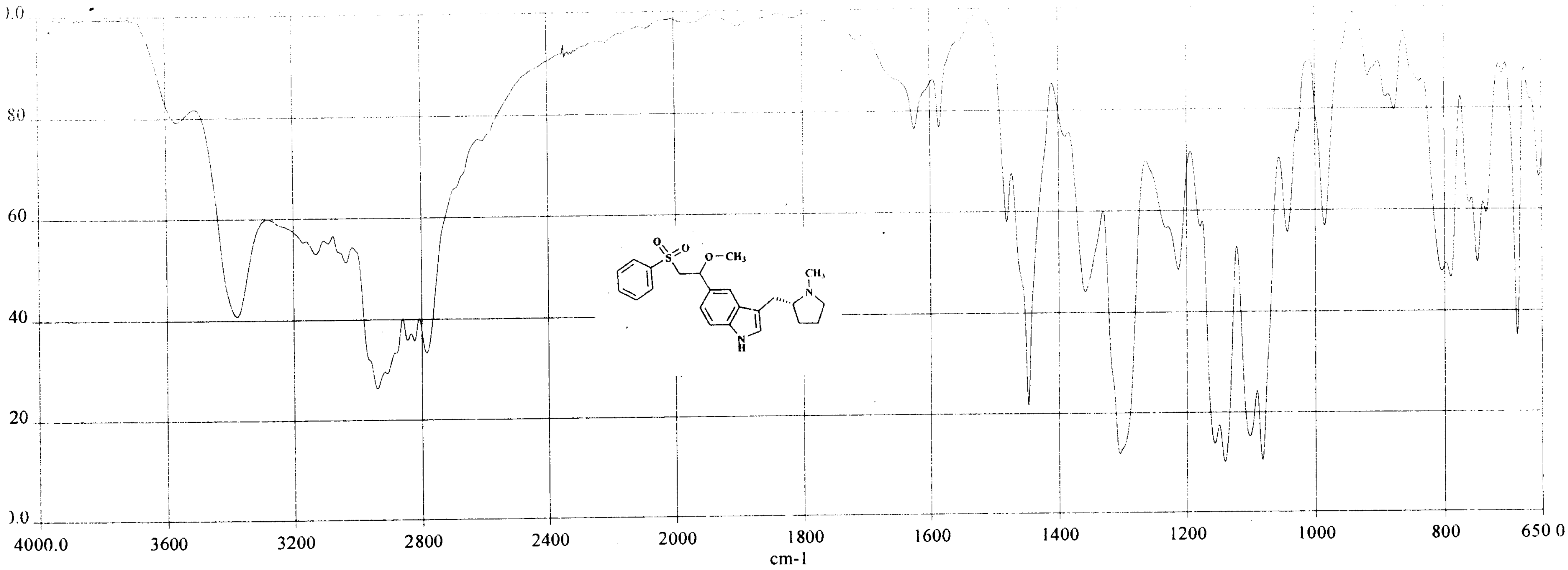

29

Status

Filename: ELETRIPTAN METHOXY IMPURITY-KSA(1637)66.002
Date Created: Saturday, March 17, 2012 3:14 PM India Standard Time
Analyst: Analyst
Description: B.NO:KSA(1637)66
Comments:
SAMPLE:ELETRIPTAN METHOXY IMPURITY
PROJECT:ELETRIPTAN HBR
2.25 mg of sample in 455 of KBr

Abscissa: (cm-1)
Start: 4000.00
End: 450.00
Interval: -1.000000

Ordinate: (%T)
Maximum: 100.00
Minimum: 10.00
Points: 3551

Instrument Model : **Spectrum One**
Instrument Serial Number : 53009

ELETRIPTAN METHOXY IMPURITY-KSA(1637)66.pk

ELETRIPTAN METHOXY IMPURITY-KSA(1637)66.002 3351 4000.00 650.00 10.00 99.60 4.00 %T 8 1.0(
B.NO:KSA(1637)66
REF 4000 99.51 2000 98.38 600
3563.14 79.14 3379.40 40.82 3130.25 53.04 3037.14 51.34 2938.62 26.29
2844.18 35.85 2822.47 35.71 2785.17 33.29 2343.57 91.20 1900.10 97.12
1624.30 76.53 1585.29 76.70 1480.43 57.93 1446.98 21.70 1358.37 44.17
1303.88 11.79 1213.28 48.41 1178.45 56.78 1156.16 13.76 1140.80 9.98
1102.30 15.12 1082.57 10.35 1042.55 55.65 985.34 56.81 918.23 86.53
877.02 79.70 804.07 47.93 789.90 46.72 748.06 49.65 734.05 59.10
709.69 86.87 687.95 35.12 654.16 66.42

APL RESEARCH CENTRE
ANALYTICAL RESEARCH DEPARTMEN'

INSTRUMENT ID : RCII_AE088

PROJECT NAME : MAR_2012\RCII_AE088

Sample ID	:	#KSA(1637)66/Eletriptan Methoxy imp	Proc. Chnl. Descr :	W2996 PDA 225.0 nm [illegible]
			Date Acquired :	3/29/2012 4:00:13 PM IST
Run Time	:	60.0 Minutes	Acq. Method Set :	Eletriptan_RS_MET
Vial	:	27		
Injection	:	1	Date Processed :	3/30/2012 5:18:55 PM IST
Injection Volume	:	20.00 ul	Processing Method :	Eletriptan_RS_Pro

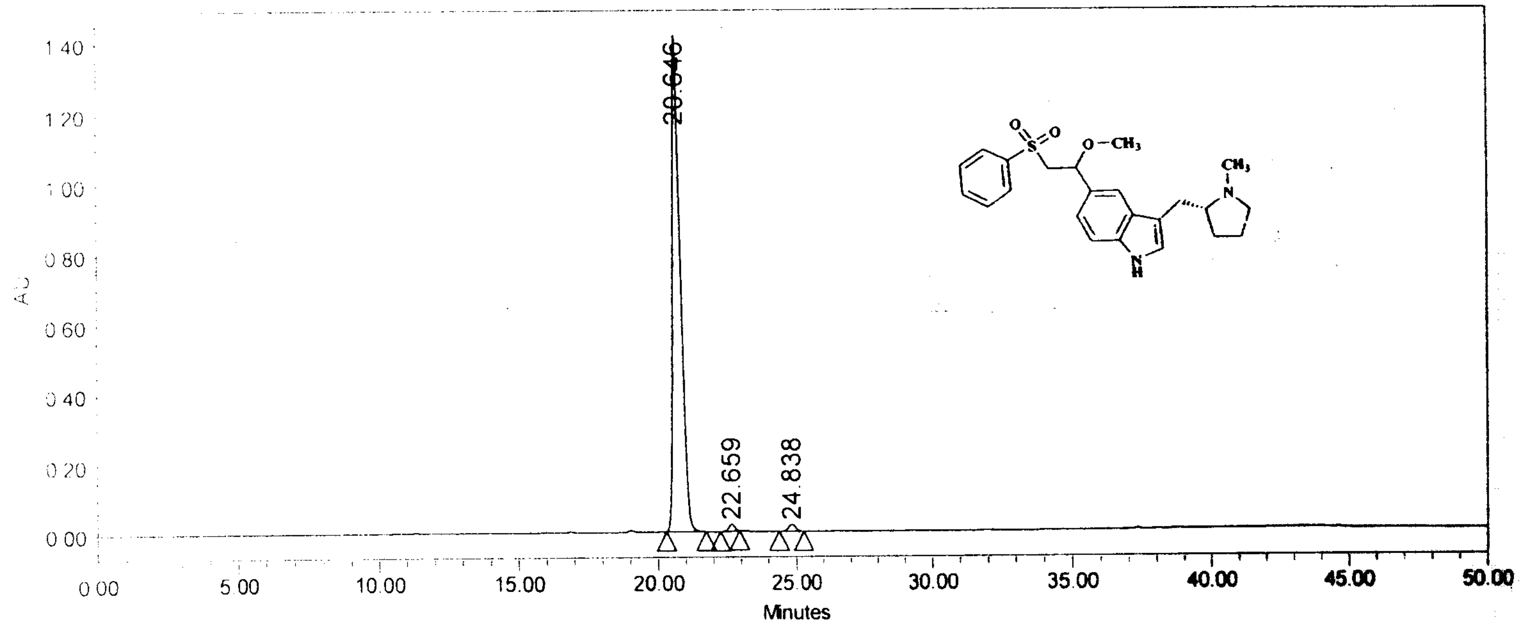

Peak Results

	RT (min)	Area (µV*sec)	% Area	RT Ratio	Name
1	20.65	28054773	97.66		Eletriptan Methoxy Imp
2	22.66	298205	1.04	1.10	Peak2
3	24.84	374071	1.30	1.20	Peak3

(ELETRIPTAN TETRACYCLIC IMPURITY)

SAMPLE: msk-1739-141

Eletriptan Unk Imp
msk-1739-141

Solvent: dmso
Temp. 25.0 C / 298.1 K
Operator: apl
File: PROTON_01
VNMRS-500 "APLV500"

PULSE SEQUENCE
Relax. delay 1.000 sec
Pulse 45.0 degrees
Acq. time 2.045 sec
Width 8012.8 Hz
32 repetitions

OBSERVE H1, 499.8205833

DATA PROCESSING
FT size 32768
Total time 1 minutes

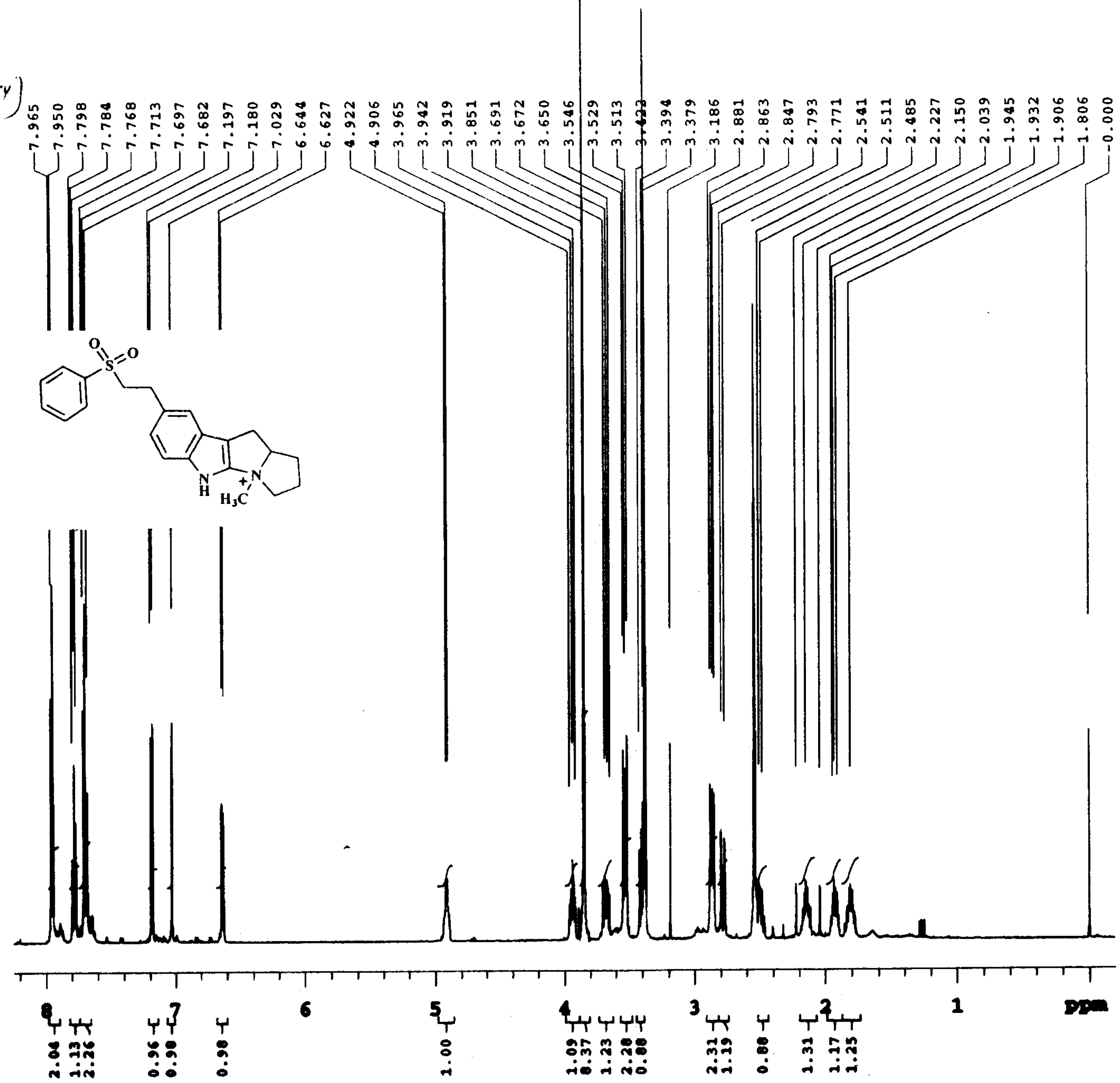

31

[redacted]ys/data/2011/Jun | Sample: msk-1739-141_20110618 | File: PROTON_01

(ELETRIPTAN TETRACYCLIC IMPURITY)

SAMPLE: msk-1739-141

Eletriptan unk Imp
msk-1739-141

Solvent: dmso
Temp. 25.0 C / 298.1 K
Operator: apl
File: dept_01
VNMRS-500 "APLV500"

PULSE SEQUENCE: DEPT
Relax. delay 1.000 sec
Pulse 90.0 degrees
Acq. time 1.022 sec
Width 31250.0 Hz
1920 repetitions

OBSERVE C13, 125.6799918
DECOUPLE H1, 499.8231069
Power 39 dB
on during acquisition
off during delay
WALTZ-16 modulated

DATA PROCESSING
Line broadening 0.5 Hz
FT size 65536
Total time 64 minutes

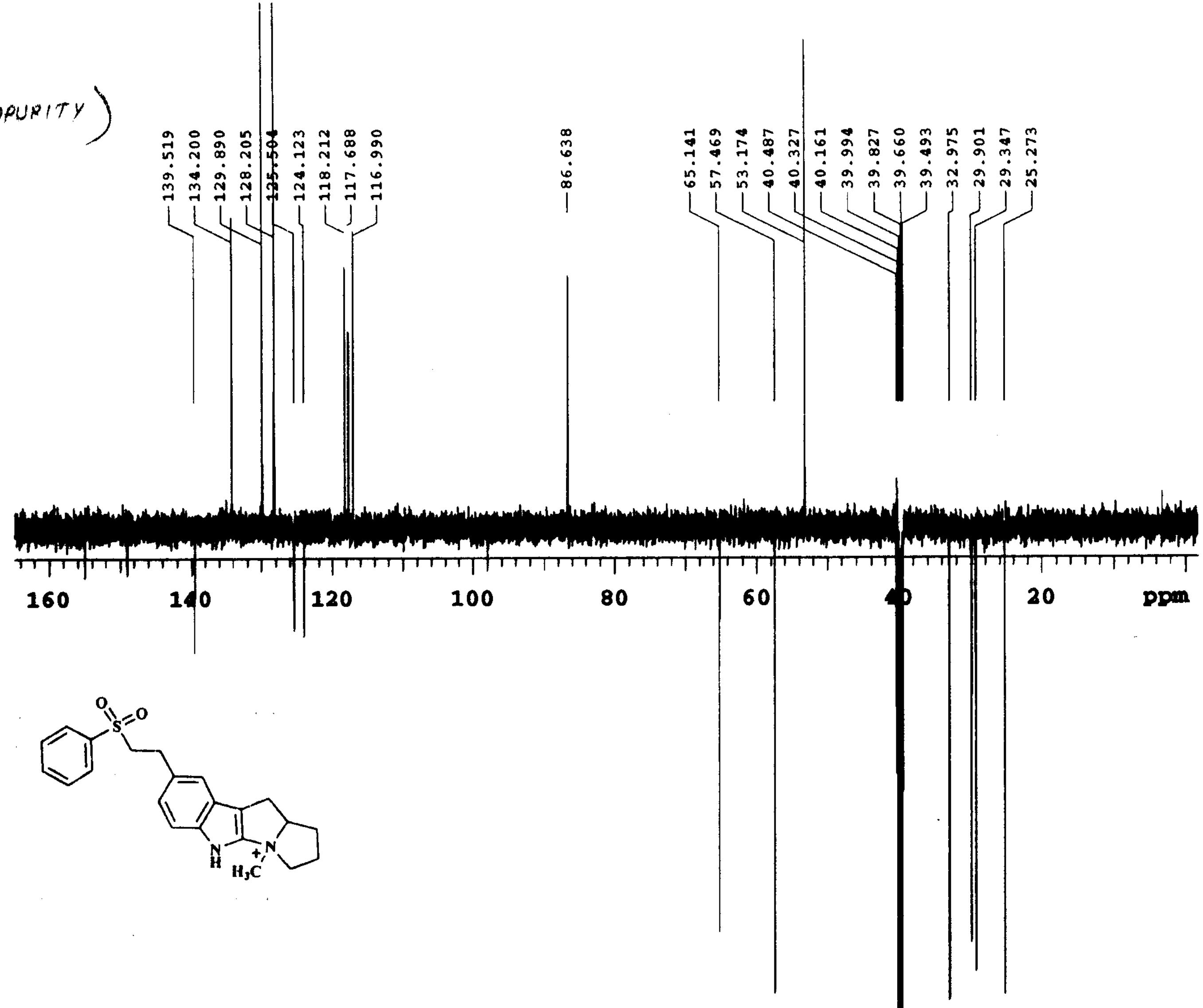

32

A[redacted]ys/data/2011/Jun | Sample: msk-1739-141_20110617 | File: dept_01

[illegible]

ELETRIPTAN UNKNOWN IMPURTY

Injection Date	: 6/18/2011 6:03:48 PM	Seq. Line	: 2
Sample Name	: MSK(1739)141	Location	: Vial 6
Acq. Operator	: MSR/KPR	Inj	: 1
Acq. Instrument	: Instrument 1	Inj Volume	: 10 µl
Different Inj Volume from Sequence !		Actual Inj Volume	: 5 µl
Acq. Method	: C:\HPCHEM\1\METHODS\POS_NEG.M		
Last changed	: 6/18/2011 5:42:15 PM by MSR/KPR		
Analysis Method	: C:\HPCHEM\1\METHODS\POS_NEG.M		
Last changed	: 7/8/2011 4:23:52 PM by MSR/KPR		

POS-NEG METHOD.

(ELETRIPTAN TETRACYCLIC IMPURITY)

MS Spectrum

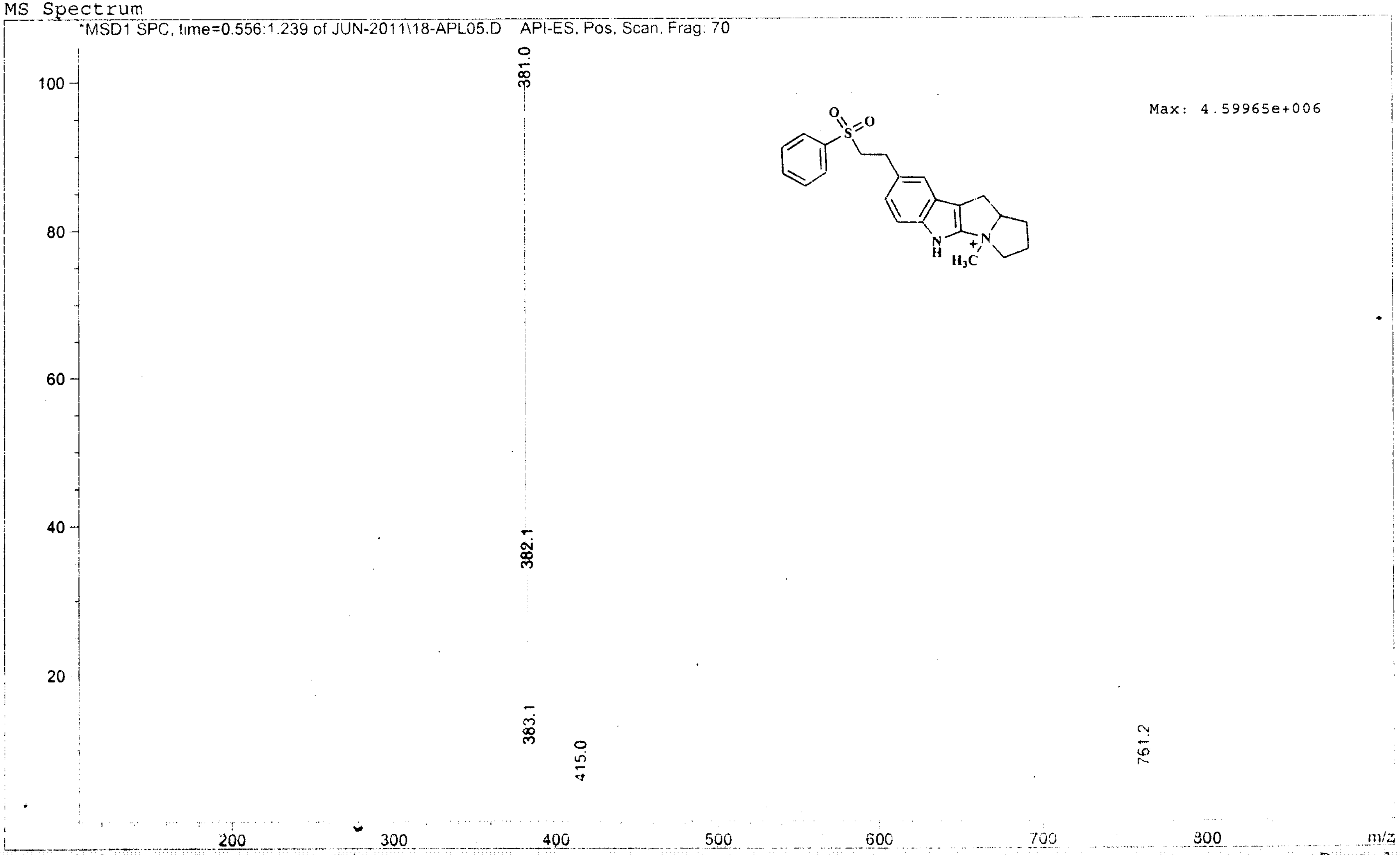

33

APL RESEARCH CENTRE RCII_AE009

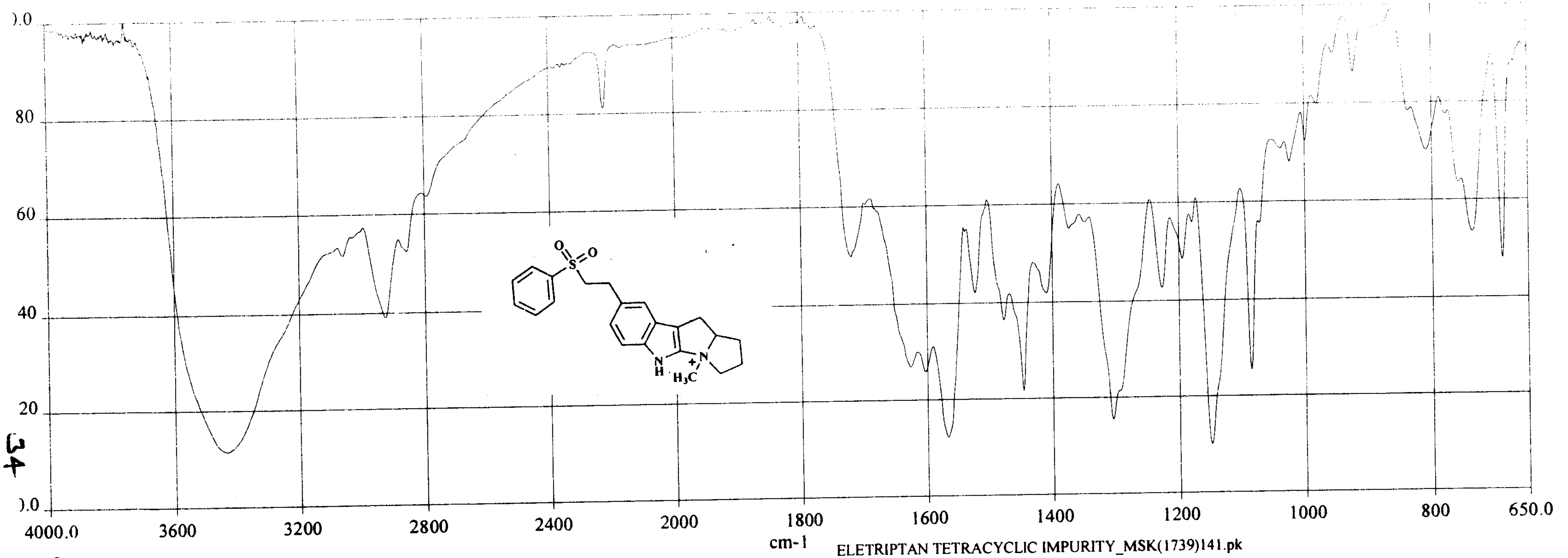

ELETRIPTAN TETRACYCLIC IMPURITY_MSK(1739)141.pk

Status

Filename: ELETRIPTAN TETRACYCLIC IMPURITY_MSK(1739)141.001
Date Created: Wednesday, March 14, 2012 6:15 PM India Standard Time
Analyst: Analyst
Description: B.NO:MSK(1739)141 A.R.NO:S-1202058
Comments:
SAMPLE:ELETRIPTAN TETRACYCLIC IMPURITY
PROJECT:ELETRIPTAN HBR
2.01 mg OF SAMPLE IN 410.00 mg OF KBr

Abscissa: (cm-1)
Start: 4000.00
End: 450.00
Interval: -1.000000

Ordinate: (%T)
Maximum: 100.00
Minimum: 10.00
Points: 3551

Instrument Model : Spectrum One
Instrument Serial Number : 53000

ELETRIPTAN TETRACYCLIC IMPURITY_MSK(1739)141.001 3351 4000.0 650.0 10.0 100.0 4.0 %T 8 1.[illegible]
B.NO:MSK(1739)141 A.R.NO:S-1202058
REF 4000 98.6 2000 95.2 600
3925.0 96.8 3880.0 95.7 3835.5 96.7 3815.5 96.3 3779.3 95.3
3436.5 11.3 3062.6 51.5 2926.4 39.1 2857.3 52.4 2225.9 81.1
1907.0 96.1 1826.9 97.1 1719.3 49.8 1626.7 27.0 1603.0 25.9
1567.4 12.1 1523.1 42.0 1478.0 36.4 1446.8 21.6 1409.4 41.8
1373.0 54.9 1348.3 56.2 1305.0 15.3 1226.7 42.6 1194.6 48.5
1178.7 55.9 1148.9 9.9 1085.6 25.4 1038.0 70.8 1023.8 68.1
998.6 72.3 980.6 79.9 954.5 90.1 923.0 86.3 808.8 70.2
737.1 53.4 689.4 48.0

14/3/12

APL RESEARCH CENTRE
ANALYTICAL RESEARCH DEPARTMEN'

INSTRUMENT ID RCII_AE088

PROJECT NAME : MAR_2012\RCII_AE088

Sample ID	MSK(1739)141/Eletriptan Tetra Cyclic imp	Proc. Chnl. Descr	:	W2996 PDA 225.0 nm
Run Time	60.0 Minutes	Date Acquired	:	3/29/2012 5:03:43 PM IST
Vial	28	Acq. Method Set	:	Eletriptan_RS_MET
Injection	1	Date Processed	:	3/30/2012 5:20:17 PM IST
Injection Volume	20.00 ul	Processing Method	:	Eletriptan_RS_Pro

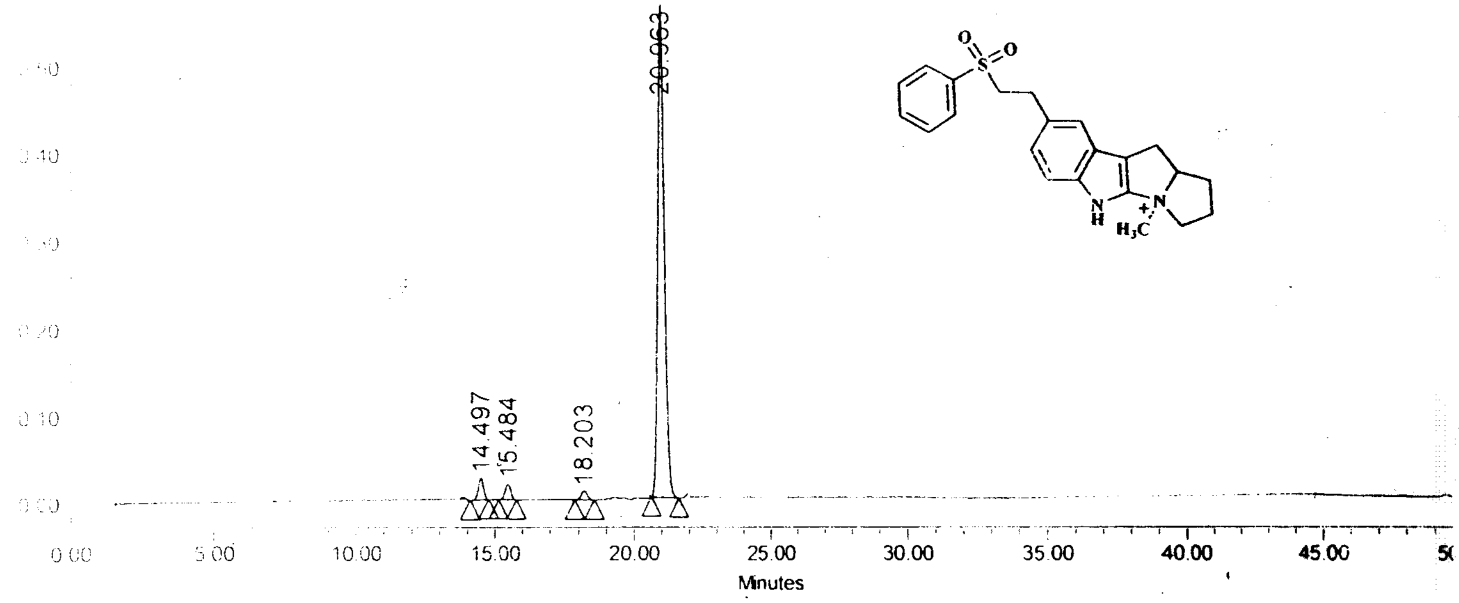

Peak Results

	RT (min)	Area (µV*sec)	% Area	RT Ratio	Name
1	14.50	342690	3.43	0.69	Peak3
2	15.48	252697	2.53	0.74	Peak4
3	18.20	163072	1.63	0.87	Peak5
4	20.96	9225094	92.40	1.00	Eletriptan Tetra Cyclic imp

APL RESEARCH CENTRE-II
ANALYTICAL RESEARCH DEPARTMENT

INSTRUMENT ID RCII_AE155

PROJECT NAME APR_2012\RCII_AE155

Sample ID:	Imp Mix		
Vial:	11	Proc. Chnl. Descr.:	Detector A 225nm
		Date Acquired:	4/12/2012 11:53:49 AM IST
Injection :	1	Acq. Method Set:	Eletriptan_RS_MET
Injection Volume:	20.00 ul	Date Processed:	4/13/2012 9:35:28 AM IST
Run Time:	60.0 Minutes	Processing Method:	Eletriptan_RS_Proc1

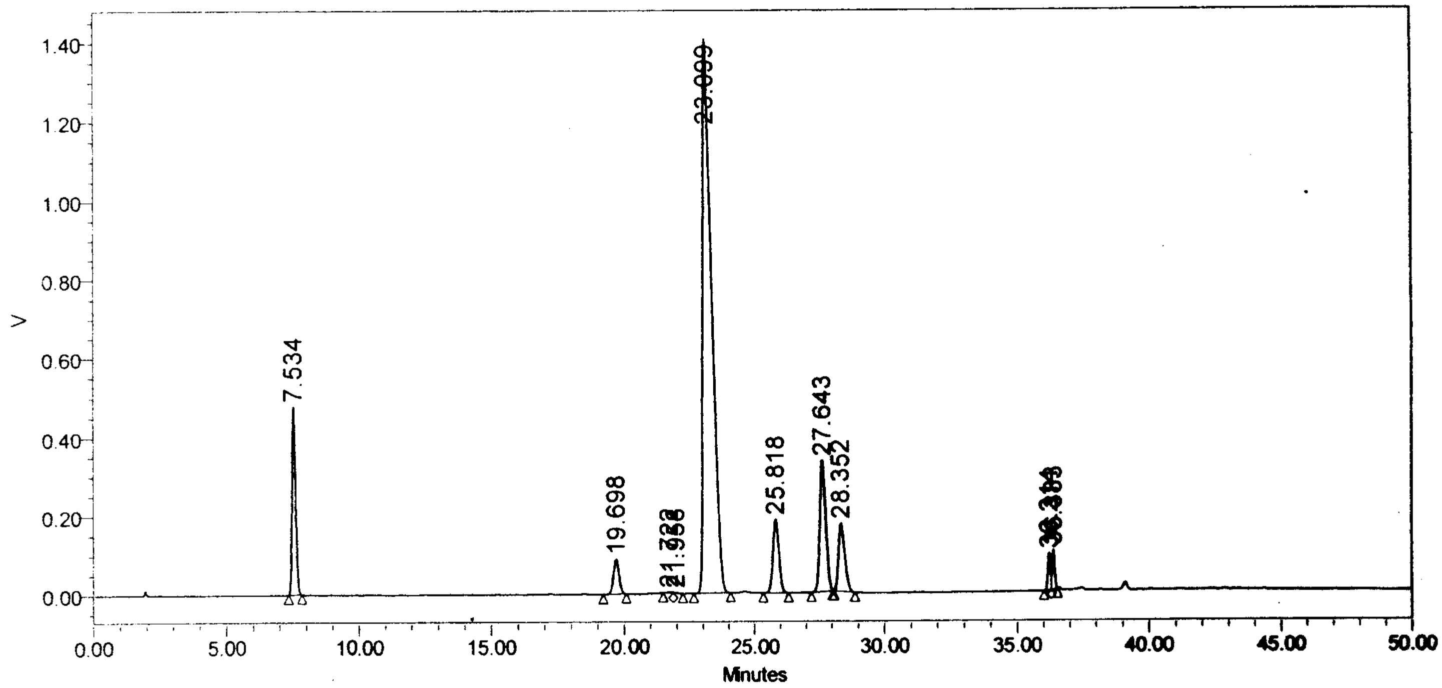

PEAK RESULTS

	RT	Area (µV*sec)	% Area	RT Ratio	Compound Name	Compound Label
1	7.53	4543538	8.76	0.33	Desbromo Indole Pyrrolidine imp	6
2	19.70	1290120	2.49	0.85	Ethyl Indole Pyrrolidine imp	5
3	21.72	39054	0.08	0.94	Eletriptan Methoxy imp	7
4	21.96	30510	0.06	0.95	Eletriptan Tetracyclic imp	8
5	23.10	33414027	64.39	1.00	Eletriptan	1
6	25.82	2981761	5.75	1.12	Eletriptan N-Oxide-1	3
7	27.64	5416067	10.44	1.20	Eletriptan N-Oxide-2	4
8	28.35	2867398	5.53	1.23	Des acetyl ensulphone derivative	13
9	36.21	642276	1.24	1.57	Eletriptan Dimer	2
10	36.38	665175	1.28	1.58	Eletriptan Dimer	2
